# Conformational analysis and interaction of the *Staphylococcus aureus* transmembrane peptidase AgrB with its AgrD propeptide substrate

**DOI:** 10.3389/fchem.2023.1113885

**Published:** 2023-05-05

**Authors:** Philip Bardelang, Ewan J. Murray, Isobel Blower, Sara Zandomeneghi, Alice Goode, Rohanah Hussain, Divya Kumari, Giuliano Siligardi, Katsuaki Inoue, Jeni Luckett, James Doutch, Jonas Emsley, Weng C. Chan, Philip Hill, Paul Williams, Boyan B. Bonev

**Affiliations:** ^1^ Biodiscovery Institute and School of Life Sciences, University of Nottingham, Nottingham, United Kingdom; ^2^ School of Biosciences, University of Nottingham, Loughborough, United Kingdom; ^3^ Diamond Light Source Ltd, Harwell Science and Innovation Campus, Didcot, Oxfordshire, United Kingdom; ^4^ ISIS Neutron and Muon Source, Rutherford Appleton Laboratory, Harwell Oxford, Didcot, United Kingdom; ^5^ School of Pharmacy, Biodiscovery Institute, University of Nottingham, Nottingham, United Kingdom

**Keywords:** quorum sensing, AgrB, AgrD, *Staphylococcus aureus*, membrane protein structure, molecular dynamics simulations, synchrotron radiation circular dichroism, small angle X-ray scattering

## Abstract

Virulence gene expression in the human pathogen, *S. aureus* is regulated by the *agr* (accessory gene regulator) quorum sensing (QS) system which is conserved in diverse Gram-positive bacteria. The *agr* QS signal molecule is an autoinducing peptide (AIP) generated via the initial processing of the AgrD pro-peptide by the transmembrane peptidase AgrB. Since structural information for AgrB and AgrBD interactions are lacking, we used homology modelling and molecular dynamics (MD) annealing to characterise the conformations of AgrB and AgrD in model membranes and in solution. These revealed a six helical transmembrane domain (6TMD) topology for AgrB. In solution, AgrD behaves as a disordered peptide, which binds N-terminally to membranes in the absence and in the presence of AgrB. *In silico*, membrane complexes of AgrD and dimeric AgrB show non-equivalent AgrB monomers responsible for initial binding and for processing, respectively. By exploiting split luciferase assays in *Staphylococcus aureus*, we provide experimental evidence that AgrB interacts directly with itself and with AgrD. We confirmed the *in vitro* formation of an AgrBD complex and AIP production after Western blotting using either membranes from *Escherichia coli* expressing AgrB or with purified AgrB and T7-tagged AgrD. AgrB and AgrD formed stable complexes in detergent micelles revealed using synchrotron radiation CD (SRCD) and Landau analysis consistent with the enhanced thermal stability of AgrB in the presence of AgrD. Conformational alteration of AgrB following provision of AgrD was observed by small angle X-ray scattering from proteodetergent micelles. An atomistic description of AgrB and AgrD has been obtained together with confirmation of the AgrB 6TMD membrane topology and existence of AgrBD molecular complexes *in vitro* and *in vivo*.

## Introduction

*Staphylococcus aureus* is an important human pathogen capable of causing a broad range of mild to severe infections from, for example, wound, blood-borne and respiratory infections to exotoxin-mediated diseases such as scalded skin and toxic shock syndromes (Rasigade et al., 2014; Balasubramanian et al., 2017; Cheung et al., 2021). The treatment and management of such infections has been compounded by the emergence of multi-antibiotic resistant strains such as MRSA (methicillin resistant *Staphylococcus aureus*) in both hospital and community settings highlighting the urgent need for novel anti-infective agents (Turner et al., 2019; Cheung et al., 2021).

The pathogenesis of *S. aureus* infections depends on the regulated production of diverse cell wall associated colonization factors and tissue damaging exotoxins and exoenzymes (Balasubramanian et al., 2017; Cheung et al., 2021). These are co-ordinately controlled via bacterial cell-to-cell communication or quorum sensing (QS) in concert with bacterial cell population density. In *S. aureus,* QS depends on the *agr* (accessory gene regulator) system which represses genes coding for cell surface protein colonization factors, such as the immunoglobulin-binding, Protein A and the fibronectin-binding proteins FnBPA and FnBPB while activating expression of the genes for secreted exotoxins such as α-haemolysin (Jenul and Horswill, 2019). In experimental animal models of *S. aureus* infection, *agr* mutants exhibit significantly reduced virulence, highlighting a key role for this regulatory locus in staphylococcal disease. Hence the *agr* system has considerable potential as a target for novel anti-infective agents that prevent infection by attenuating virulence (Gordon et al., 2013).

The *agr* locus consists of two divergent transcriptional units, *agrBDCA* and the regulatory RNA effector, RNAIII. AgrA and AgrC constitute a two-component system (TCS) in which the transmembrane AgrC is the sensor kinase and cytoplasmic AgrA is the response regulator (Wang et al., 2015a; Jenul and Horswill, 2019). In the *agr* system, the diffusible QS signalling molecule is a peptide thiolactone (the autoinducing peptide, AIP), in which the thiol moiety within the central cysteine residue is covalently linked to the C-terminal amino acid carboxylate, forming a cyclic thioester. *Staphylococcus aureus* strains can be divided into four different *agr* groups based on their ability to cross-activate or inhibit *agr* expression (Ji et al., 1995; Ji et al., 1997; McDowell et al., 2001; Jensen et al., 2008). AIP-1 for example, is produced from the AgrD1 pro-peptide via AgrB1 and sensed by AgrC1 but is a competitive inhibitor of the AIP-2/AgrC2 interaction. Once an AIP reaches a critical extracellular concentration, it binds to and activates its cognate AgrC receptor which in turn phosphorylates AgrA. This binds to the *agrBDCA* P2 and P3 promoters inducing a positive-feedback circuit that autoinduces AIP synthesis and drives virulence factor production directly via AgrA or via the AgrA-dependent effector RNAIII (Wang et al., 2015a; Jenul and Horswill, 2019).

**FIGURE 1:**
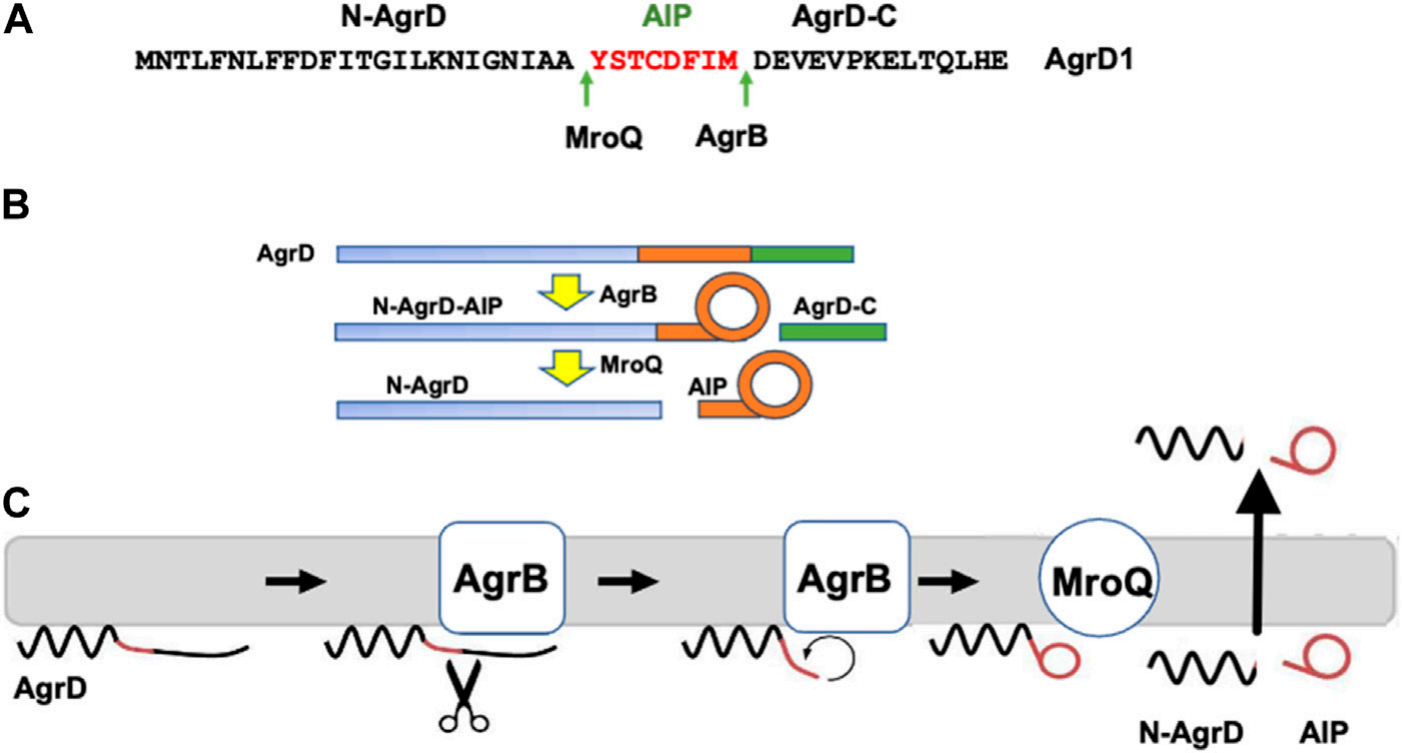
Processing of the AgrD pro-peptide to generate the active cyclic AIP signal molecule. **(A)** Amino acid sequence of AgrD1 showing the AgrB1 and MroQ cleavage sites. **(B)** Schematic highlighting the processing of AgrD by AgrB and MroQ to release N-AgrD and the cyclic AIP. **(C)** Schematic showing the formation and release of the AIP and N-AgrD at the cytoplasmic membrane. Cleavage of AgrD by AgrB releases a 14 amino acid C-terminal peptide (AgrD-C) which is degraded in the cytoplasm. N-AgrD-AIP is cleaved by MroQ to release N-AgrD and the mature AIP. The mechanism by which the AIP and N-AgrD are exported is not known.

AIPs are derived from the proteolytic processing of AgrD and exported from the cell to the extracellular environment although at present the AIP transport mechanism is not understood (Wang et al., 2015a). AgrD is composed of an N-terminal amphipathic leader (N-AgrD (24-25 amino acids), a mid-region of 7-9 amino acid residues that constitutes the AIP and a charged C-terminal tail (AgrD-C; 14-15 amino acids; Figure 1A). The generation of extracellular AIP requires 4 membrane-associated steps (Figures 1B,C): i) removal of AgrD-C, ii) formation of the thiolactone macrocycle, iii) removal of N-AgrD and iv) AIP and N-AgrD export. These steps involve transmembrane peptidases including the cysteine-protease AgrB which is required for steps i) and ii), whereas step iii) depends on MroQ and potentially signal peptidase 1 (SpsB) (Qiu et al., 2005; Kavanaugh et al., 2007; Wang and Muir Tom, 2016; Zhao et al., 2022) (Figure 1C). Efficient AIP production is driven by stabilization of the macrocycle via the membrane association of the thiolactone-containing intermediate and by the rapid degradation the AgrD-C fragment (Wang et al., 2015b).

As yet, no high-resolution structures of AgrB proteins are available as molecular and structural analysis of poorly behaved recombinant proteins is complicated by the need to maintain a folded state in membranes. However, topology predictions and mapping based on site specific mutagenesis and substituted cysteine accessibility assays have suggested two different and somewhat contradictory AgrB models–a 6 transmembrane domain (TMD) model with both termini at *cis* (Qiu et al., 2005) and a 4 TMD with an additional half-transmembrane hairpin where both termini are at *trans* (Thoendel and Horswill, 2013).

To begin resolving these contradictions and gain further insights into the structure of AgrB and its interactions with substrate AgrD, we combined homology modelling with molecular dynamics (MD) simulations to construct an all-atom 3D model specifically for orthologues AgrB1 and AgrD1 which we will generally refer to here as AgrB and AgrD. The formation of molecular complexes between AgrB and AgrD have been inferred from function (Wang et al., 2015a; Wang et al., 2015b) but not observed directly. We therefore complemented computational modelling with cellular, biochemical and biophysical approaches and propose a model incorporating an AgrB dimer in which one monomer facilitates insertion and positioning of AgrD in the correct orientation for catalytic processing by the second AgrB monomer.

## Materials and methods

### Homology modelling and molecular dynamics simulations

The amino acid sequence (https://www.uniprot.org/uniprotkb/P0C1P7/entry) for the *S. aureus agr* group 1 AgrB1 protein was obtained from Uniprot (Consortium, 2020) and the fold modelled using the LOMETS server (Wu and Zhang, 2007). The top scoring model has six transmembrane domains (6TMD) topology and agreed well with secondary structure estimate from JPred (Drozdetskiy et al., 2015). Top three templates include the K-channel TREK2 (4BW5) (Dong et al., 2015), Z-score = 1.14, multidrug transporter MATE (3VVN) (Tanaka et al., 2013), Z-score = 1.03, and the intramembrane protease Rce1 (4CAD) (Manolaridis et al., 2013), Z-score = 1.19. Molecular complexes between AgrB and AgrD, as well as (AgrB)_2_AgrD, were built using CluPro (Kozakov et al., 2017) ahead of integration into simulated membranes and further MD simulations. The AgrB model was embedded into a 135 × 135Å simulated membrane patch of 1-palmitoyl-2-oleoyl-*sn*-glycero-3-[phospho-*rac*-(1-glycerol)] (POPG)/1-palmitoyl-2-oleoyl-*sn*-glycero-3-phosphatidylatidylethanolamine (POPE)/1′,3′-bis [1-palmitoyl-2-oleoyl-sn-glycero-3-phospho]-glycerol (cardiolipin, CL) in 75:20:5 molar ratio using the CHARMM 36 force-field (Lee et al., 2016) and solvated in a neutral cuboid box extending 20Å away from the solvated protein poles in *Z*-direction using CHARMM-GUI (Jo et al., 2008). Periodic boundary conditions were used with wrapping on all cuboid faces. For membrane docking experiments, AgrD was placed with edge 30 Å above the membrane surface. During docking to membranes with AgrB, AgrD was placed on the “wrong” biological side (“*trans*”) of the membrane. Without guidance, AgrD was allowed to cross the water box boundary between “*trans*” and “*cis*” (non-biological, simulation condition) to approach the correct, “*cis*,” side of the membrane in an unbiased way. The docking calculations were repeated thrice. All minimization, equilibration, and production dynamics were performed using NAMD (Phillips et al., 2005) with a time step of 2 fs. The non-bonded cut-off was 12 Å and the non-bonded neighbour list was updated at every time step. Long-range electrostatics were treated using the particle-mesh Ewald method (Darden et al., 1993). Minimization was performed using the standard NAMD minimization algorithm for 1000 steps followed by equilibration at 300K for 2500 steps in a NVT ensemble, while production runs were on NPT ensemble. Consecutive pre-production runs of 1 and 10 ns at 300K, as well as all minimisation runs, were carried out on a 1U GPGPU server with TESLA K80 and K40 GPU accelerators. The AgrB model was annealed for 300 ns of production simulations using NAMD (Phillips et al., 2005) at 50 ns consecutive blocks on the Nottingham HPC Facility, the MidPlus HPC, HPC Midlands Plus, and the 1U GPGPU server. A steady RMSD value of 2.56 Å was reached after the first 30 ns. Trajectory analysis and visualisation were performed using VMD (Phillips et al., 2005) and UCSF Chimera (Pettersen et al., 2004).

### Bacterial strains and growth conditions

The bacterial strains and plasmids used are described in Supplementary Tables S1, S3 and S4. *Escherichia coli* and *S. aureus* strains were routinely grown in lysogeny broth (*Escherichia coli*) or tryptic soy broth (TSB) respectively at 37°C with shaking at 200rpm.

### Cloning and expression of AgrB

Unless otherwise stated *agrB1* expression constructs were generated using NEBuilder HiFi DNA assembly as described by the manufacturer (New England Biolabs). Briefly, *agrB1* from *S. aureus* strain JE2 (Supplementary Table S1) was amplified using primer pair IB1 and IB2 (Supplementary Table S2). Inverse PCR was used to amplify the expression construct pCDFDuet-1 [Takara (Supplementary Table S1)] using primer pair IB3 and IB4 (see Supplementary Table S2). The PCR products were added to the HiFi reaction mixture and incubated at 50°C for 30 min. Post incubation, ligation mixtures were desalted and transformed into electrocompetent *E. coli*, DC10B (Supplementary Table S1). All DNA constructs were confirmed by DNA sequencing (Source Biosciences) using inhouse commercial primers specific for the pCDF-Duet-1 vector.

The *agrB1* expression plasmid, pCDFDuet-*agrB1* (Supplementary Table S1) was transformed into *E. coli* C41 (DE3) and cultures grown overnight in Terrific Broth (TB) containing 100 μg/mL spectinomycin. Bacterial cells were diluted 1 in 200 in 30 mL TB and grown to OD_600_ 0.8-1.0. IPTG (0.5 mM) was added and cells were grown for a further 3 h at 37°C. Post-induction cells were harvested by centrifugation (6000 × *g*, 10 min). Cell pellets were suspended in 1 mL buffer (20 mM Tris-HCl pH 7.5, 5 mM MgSO_4_) and were mechanically lysed using a FastPrep homogenizer. Suspensions were centrifuged at 12,00 × *g* for 2 min to remove cell debris and then at 100,000 × *g* for 1 h to pellet the cell envelope fraction. Cell envelopes were resuspended in 100 μL phosphate buffered saline (PBS), pH 7.4.

### Construction of split luciferase tagged AgrB and AgrD

To investigate AgrB and AgrBD protein-protein interactions in *S. aureus* cells, we used the Nanobit^®^ split luciferase assay (Promega) where AgrB and AgrD were tagged either N- and C- terminally or *vice versa* with one of the two separate Nanobit subunits (the small BiT (SmBiT, 1.3 kDa) or the Large BiT (LgBiT, 18 kDa) of an engineered luciferase. The tagged genes were introduced either into the ectopic *attB*2 site on the *S. aureus* chromosome or on low copy plasmids (Supplementary Table S1). As controls we also constructed pSK2-P2 plasmids containing the SmBit or LgBiT alone. Supplementary Tables S1, S3 and S4 summarize the bacterial strains and plasmids generated for these assays.

The *agrB1* gene including the upstream P2 promoter region was PCR amplified with primer pair EJM 920 and EJM 921 (Supplementary Table S2). Primer incorporated *Hind*III and *Bam*HI restriction sites were utilised to directionally clone the amplified fragment into the multiple cloning site of the low copy shuttle vector pSK5630 (Grkovic et al., 2003) generating plasmid pSK-P2-*agrB*. Protein interaction studies were based on split luciferase technology. Four codon optimised synthetic oligonucleotides were synthesized by Eurofins Genomics Ltd coding for the N-terminal LargeBiT, N-terminal SmallBiT, C-terminal LargeBiT and C-terminal SmallBiT each with a recommended 16 amino acid flexible linker. The *agrB* split luciferase gene fusions were generated by HiFi cloning (New England Biolabs Ltd). Briefly, PCR amplified split luciferase fragments were ligated to an inverse PCR generated pSK-P2-*agrB* template (see Supplementary Table S2) at either the N- or C- termini of AgrB generating four plasmid constructs, pSK-P2-NL*agrB,* pSK-P2-NS*agrB,* pSK-P2-*agrB*CL and pSK-P2-*agrB*CS. Chromosomal *agrB*-split luciferase “partner strains” were generated at the *attB2* phage integration site of strain CYL12349 as described by Lei et al. (2012). A kanamycin resistant version of the *S. aureus* integration vector pLL102, pLL102k, was generated by replacing the native tetracycline resistance cassette with the kanamycin resistance cassette from pDG729 (*Cla* I/*Hind* III fragment) (Guérout-Fleury et al., 1995). The four P2-pSKagrB/split luciferase fusions as well as the pSK-P2-*agrB* template were PCR amplified using primer pair EJM 929 and 930 (Supplementary Table S2). Using HiFi cloning (New England Biolabs Ltd), PCR generated *agrB* fusions were ligated to an inverse PCR generated DNA template of pLL102k (EJM927/928). The *agrB*-split luciferase integration vectors were transformed into *S. aureus* strain CYL12349 with selection using kanamycin (100 μg/mL). Integration at the *attB2* site was confirmed by PCR using primer pair EJM 55 and EJM 56 (Lei et al., 2012). Bacteriophage Phi 11 transduction was utilised to move the ectopic chromosomal *agrB*-split luciferase fusions to *S. aureus* strain SH1000 generating five possible partners, i.e., SH1000, SH1000 attB2 SK-P2-*agrB,* SH1000 attB2 SK-P2-NL*agrB,* SH1000 attB2 SK-P2-NS*agrB,* SH1000 attB2 SK-P2-*agrB*CL*,* SH1000 attB2 SK-P2-*agrB*CS (Supplementary Table S1).

To generate pSK-P2-*agrD*-split luciferase expression constructs, inverse PCR using the primers shown in Supplementary Table S2 were used to amplify the four *agrB1*-split luciferase constructs described above. Briefly, inverse PCR was used to generate an *agrB1* deletion and HiFi cloning (New England Biolabs Ltd) to ligate PCR-amplified *agrD*1 with primer incorporated complementary 5′-3′ sequence overhangs so that *agrD1* replaced *agrB1*. The following *agrD* plasmids (Supplementary Table S1) were obtained*,* pSK-P2-NL*agrD,* pSK-P2-NS*agrD* and pSK-P2-*agrD*CL. We were unable to generate pSK-P2-*agrD*CS.

### AgrB and AgrBD protein-protein interactions in *Staphylococcus aureus*

To investigate AgrB interactions in bacterial cells, the four plasmids pSK-P2-NL*agrB1,* pSK-P2-NS*agrB1,* pSK-P2-*agrB1*CL and pSK-P2-*agrB1*CS were transformed into each of the *S. aureus* SH1000 partner strains resulting in 16 possible combinations (Supplementary Table S3). For AgrBD interactions, the *agrD* containing plasmids were each transformed into the four *S. aureus* strains harbouring chromosomal copies of the tagged *agrB*-split luciferases (Supplementary Table S4). Antibiotic selection (kanamycin, 50 μg/mL and chloramphenicol, 5 μg/mL) was maintained during overnight culture in TSB at 37°C with shaking at 200 rpm. Bacterial cells were washed three times in TSB to remove accumulated AIP signal molecules and diluted 300-fold into fresh TSB. Cells were grown until an OD_600_ of 1.0 at which point 100 μL of cell suspension were added to white, opaque 96 well plates and Nano-Glo^®^ live cell assay reagent (Promega) added as described by the manufacturer. Bioluminescence output was quantified using a Tecan 200 pro plate-reader.

### Expression and purification of recombinant AgrB

An *E. coli* expression vector for *S. aureus* AgrB1 (pCOLD1-*agrB1*) was constructed by insertion of a synthetic *agrB1* gene (GE Healthcare; codon optimised for *E. coli*) into the *Nde*I and *Xho*I restriction sites of plasmid pCOLD1 (Takara). In brief, *E. coli* strain C41 (DE3) pCOLD1-*agrB1* was cultured at 37°C in LB medium supplemented with chloramphenicol 15 μg/mL and carbenicillin 75 μg/mL until mid-log phase and induced for expression at 16°C using 0.5 mM IPTG. After 20 h induction, the biomass was harvested by centrifugation, washed, and suspended in 20 mM Tris-HCl pH7.4, 5 mM MgCl_2_ and the cells lysed by sonication on ice. The *E. coli* membrane fraction was isolated by ultracentrifugation for 1 h at 200,000 *g* and membrane proteins extracted by incubation at 30°C, 200 rpm for 4 h in 2% (w/w) DDM (n-dodecyl-β-D-maltoside) in 20 mM sodium phosphate pH 7.4, 100 mM NaCl. Proteodetergent micelles recovered after ultracentrifugation for 25 min at 100,000 *g* were mixed overnight at 4°C with 0.5 mL of nickel-charged IMAC resin (Qiagen Ni-NTA Fast Start Kit) in 0.5% (w/w) DDM, 20 mM sodium phosphate pH 7.4, 100 mM NaCl. Contaminants were washed from the column with 20 mM sodium phosphate pH 7.1, 0.4% (w/w) DDM, 4 mM BME (β−mercaptoethanol) and 100 mM NaCl prior to elution from the column in 2 mL wash buffer supplemented with 0.6 M imidazole and 1 mM TCEP. Salts were removed using PD-10 prepacked columns (GE healthcare) through wash with buffer containing 0.4% (w/w) DDM, KHPO_4_ pH 7.4, 100 mM NaCl, 0.5 mM EDTA. AgrB protein was quantified using a NanoDrop spectrophotometer and concentration calculated based on *e* = 18,910M^-1^cm^-1^ and aliquots were stored at −80°C prior to use. AIP pro-peptide AgrD1 incorporating a 14 residue αT7 epitope leader tag (MASMTGGQQM GRIQMNTLFN LFFDFITGIL KNIGNIAAYS TCDFIMDEVE VPKELTQLHE; T7-AgrD1) was prepared commercially by solid phase synthesis and purchased from CS Bio (California, United States).

### Synchrotron radiation circular dichroism, SRCD

Samples for SRCD were prepared in the form of optically transparent proteodetergent DDM micelles to concentration of 0.13 mg/mL AgrB and AgrB/AgrD in molar equivalents, while the AgrD concentration was 1 mg/mL. Buffer exchange of the purified micelles containing His-tagged AgrB into SRCD assay buffer, 20 mM Tris and100 mM NaCl, 0.5% (w/w) DDM and 1 mM TCEP was achieved using a PD10 desalting column (GE Healthcare), and the quantity of AgrB measured by absorbance spectroscopy using a mole extinction coefficient of 18,910M^-1^ cm^-1^.

SRCD far UV experiments were performed using a nitrogen-flushed Module B end-station spectrophotometer at B23 Synchotron Radiation CD Beamline at the Diamond Light Source (Javorfi et al., 2010; Hussain et al., 2012) with bandwidth 1.1 nm, integration time of 1 s, 1 nm digital resolution, 39 min/min scan speed with 0.02 cm path length Suprasil cell (Hellma Ltd). The CD spectra were processed and analysed using CDApps (Hussain et al., 2015) where unfolding transition temperature *T*_*m*_ was estimated initially using a Boltzmann two state exchange model (Greenfield, 2006). Secondary structure estimation from CD spectra was carried out using CDApps using Continll algorithm (Sreerama and Woody, 2004). Tolerances in reported transition strength values of *τ*_*+*_ reflect primarily the quality of linear fit during calculations of equation of state and all *τ*_*+*_ values are reported to the corresponding degree of accuracy and number of significant figures.

### *In vitro* assay for AgrBD complex formation

An *in vitro* assay for AgrB activity was established using cell membranes prepared from *E. coli* C41 (DE3) pCDFDuet-*agrB1* or with purified recombinant AgrB protein. *Escherichia coli* membranes (10 μL) containing AgrB (or prepared from *E. coli* transformed with the empty vector pCDFDuet-1 as a control) were incubated with T7-AgrD1 (2 μM) for 30 min at 37°C in 20 mM Tris-HCl pH 7.5 containing 5 mM MgSO_4_. For assays with the purified AgrB, dioleoyl phosphatidylglycerol (DOPG; 1 mg/mL) was added to the buffer. Samples were heated to 55°C for 5 min in Novex Tris-glycine SDS sample buffer (ThermoFisher) and separated on 14% w/v Tris-glycine acrylamide gels (without SDS) (ThermoFisher). After electrophoresis, proteins were transferred to 0.2 μM PVDF membranes by Western blotting, blocked with 3% bovine serum albumin at 4°C overnight and probed with either an HA-tagged nanobody 2ABE10 raised in a llama against the purified recombinant AgrB or with a polyclonal antibody HRP conjugate to the T7 epitope on T7-AgrD1. For secondary antibody detection of the HA-tagged nanobody, an HRP conjugated anti-HA-tag monoclonal antibody (Stratech Scientific) was used. Western blots were developed using Amersham ECL Western blotting detection reagent (SLS) and Amersham hyperfilm ECL (SLS).

### AIP-1 bioreporter assay

We verified the biological activity of AgrB by monitoring AIP production. AIP-1 was detected and quantified as described before (Jensen et al., 2008; Sloan et al., 2019) using *S. aureus* ROJ143. This bioreporter is incapable of producing AIPs but emits bioluminescence in response to exogenously supplied AIP-1.

### Thermal analysis of protein unfolding

Secondary structure content, obtained from SRCD as a function of temperature, was used to define a normalized thermodynamic order parameter *s* Î (0,1), related to the helical protein content (*cf.* Hussain et al., 2018). For thermal analysis of protein stability, the free energy of the protein can be expressed in a Landau series expansion in terms of *s*, as follows:

$$G=G_{0}\left[\frac{s^{4}}{4}+\alpha \left(T-T_{c}\right)\frac{s^{2}}{2}+\beta \left({T-T}_{m}\right)s\right]$$

To obtain the conditions for system stability we minimise *G(s)*, which provides the thermodynamic coordinates of the positive spinodal *s*_*+*_,*T*_*+*_:

$$T=T_{+}+\frac{T_{m}-T_{+}}{2}\left[{3\left(\frac{s-s_{+}}{s_{+}}\right)}^{2}+{\left(\frac{s-s_{+}}{s_{+}}\right)}^{3}\right]$$

Further, linear fitting of T vs. the quantity:

$$f\left(s\right)={3\left(\frac{s-s_{+}}{s_{+}}\right)}^{2}+{\left(\frac{s-s_{+}}{s_{+}}\right)}^{3}$$

to the temperature dependence of *s(T)* allows us to obtain the equation of state for the protein system in the folded state. The normalised temperature separation between melting and spinodal temperatures, *t*_*+*_ *= 1—T*_*m*_ */T*_*+*_, where *t*_*+*_ ∈ (0, 0.5), is directly related to the transition cooperativity, where fully cooperative transition takes value of 0.5 and the transition occurring at a critical point is characterized by *t*_*+*_ = 0. This provides an absolute and quantitative scale for comparative classification of the structural stability of proteins, limited between absolute zero from below and with an upper limit set by the critical temperature of the system.

### Small angle X-Ray scattering, SAXS

All SAXS measurements were performed at Diamond Light Source (Harwell, UK) BioSAXS beamline B21 with a temperature regulated robotic sample environment (Inoue et al., 2013; Jo et al., 2018). Proteodetergent micelles in buffer solution, as used for SRCD, were exposed to the beam in a 1.6 mm diameter quartz capillary, using an Arinax (Grenoble, France) BioSAXS automated sample changer. The sample environment temperature was set to 30°C. The sample capillary was held in vacuum and cleaned between each measurement using a detergent wash/rinse/dry cycle. Samples were stored in 96-well plates at 20°C prior to loading. SAXS data was collected on an Eiger two-dimensional detector. A total of 60 frame exposures of 1 s from the sample and the corresponding buffer were averaged to produce each data set. Each frame was examined for the presence of radiation-induced sample damage and such frames were not reduced and excluded from further processed. The detector was at 3.6 m from the sample position, yielding a Q-range of 0.0045 Å−1 < 0.34 Å−1. Q = 4π.sinθ/λ, where 2θ is the scattering angle and *λ* is the wavelength, which in this case was 1 Å. Two-dimensional data reduction consisted of normalization for beam current and sample transmission, radial sector integration and background buffer subtraction and averaging. Further data processing was performed in SasView 5.0.5 (Doucet et al., 2021) (*cf.* Acknowledgements). The model used during data analysis is core shell ellipsoid and the scattering length densities (SLD) are as follows: SLD of the core (protein) 12.3 × 10^-6^/Å^2^, SLD of the shell (detergent) 8.3 × 10^-6^/Å^2^, SLD of the solvent 9.44 × 10^-6^/Å^2^. PRIMUS, part of the ATSAS suite developed by EMBL, is used for visualisation (Konarev et al., 2003).

## Results and discussion

In this study we investigated the molecular and functional association of the transmembrane endopeptidase AgrB with itself and its substrate, AgrD, the first step involved in generating AIP quorum sensing signal molecules. Evidence for self-association of AgrB is presented in which a ternary complex (AgrB)_2_/AgrD is assembled where we will refer to the AgrB dimer components as AgrB-I and AgrB-II.

### Modelling and conformational analysis of AgrB and AgrD structures

**FIGURE 2:**
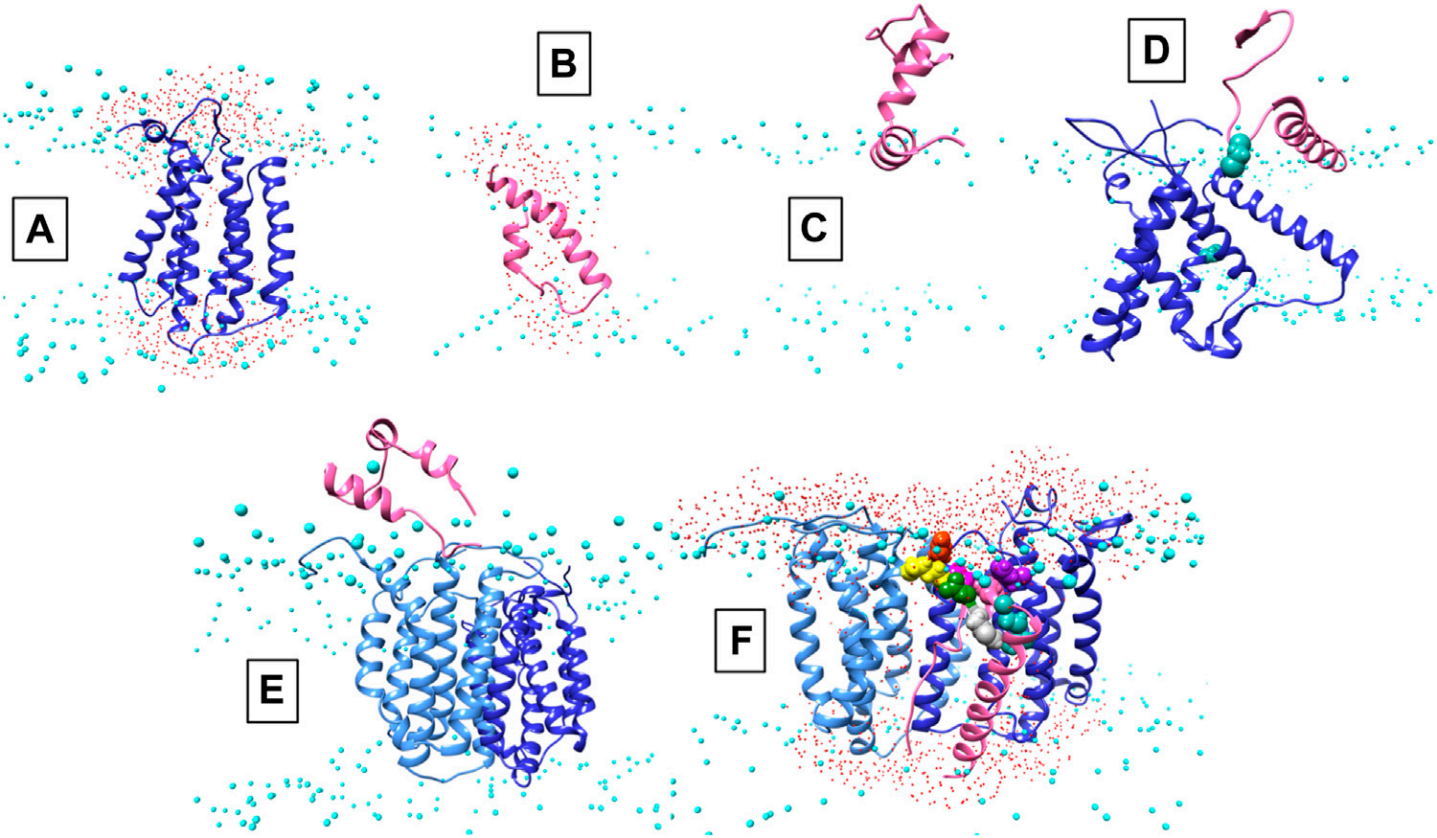
Conformations of AgrB, AgrD and their complexes in membranes. Atomistic MD simulations-annealed models of AgrD and AgrB at the end of 500 ns trajectories in hydrated membranes of POPE/POPG/CL (75/20/5): **(A)** membrane-equilibrated model of AgrB; **(B)** transmembrane conformation of AgrD; **(C)** N-terminally docked AgrD on a membrane surface; **(D)** membrane association of AgrD in the presence of AgrB showing AgrD-induced tilt on helix 5 of AgrB (*cf.* Figure 3B); **(E)** peripheral association of AgrD with integral membrane AgrB_2_ dimer; **(F)** AgrD in membrane complex with (AgrB)_2_ driven by AgrD D33/AgrB K139 contact (*cf.* Figure 3); AgrD-C28 now faces AgrB-II-C84; Phosphorus atoms of lipid phosphates are shown in cyan to mark the location of the membrane surface; water oxygens in red highlight water ingress into the membrane around protein complexes; AgrD is in pink, AgrD-C28 is shown in forest green; AgrB-I in the dimer is in cornflower blue and the second monomer AgrB-II is in blue. The cytosol side is shown up and extracellular down to provide a familiar geometry for following peptide binding to membranes and to show better details from the binding site on AgrB for AgrD.

**FIGURE 3:**
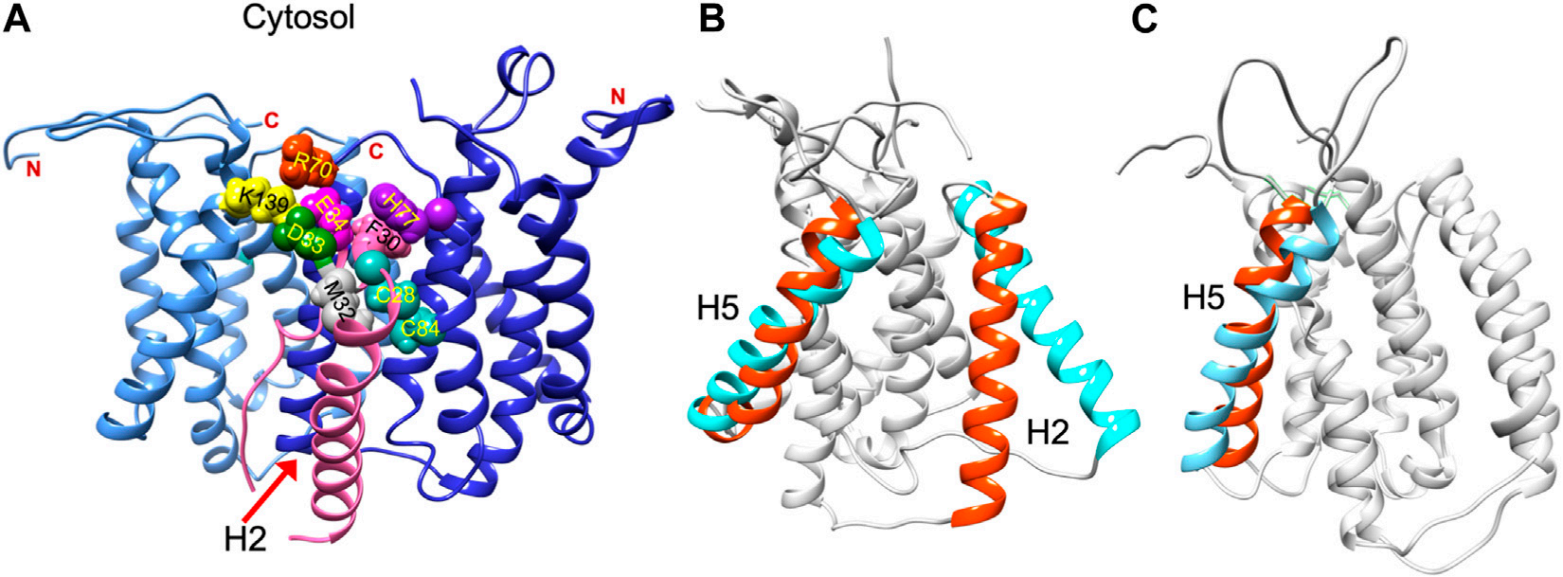
Residue contacts in the AgrB_2_/AgrD membrane complex and substrate-driven conformational change in AgrB. Conformation of the (AgrB)_2_/AgrD complex at the end of 500 ns atomistic MD simulations-annealed trajectories in hydrated membranes of POPE/POPG/CL (75/20/5) membranes, showing key residues involved in AIP pre-processing **(A)**. The two inequivalent AgrB molecules, responsible for AgrD insertion (I, cornflower blue) and catalysis (II, blue) stabilise orientation of AgrD presenting C28 and M32 close to each other and to catalytic C84 of AgrB_2_ via AgrB_1_-K139-D33 and AgrB-I R70-E34 contacts with AgrD, and *via* AgrB-II H77-F30 π-interactions. Key residues are shown in aqua (C28, C84 AgrB-II), grey (M32 AgrD), pink (F30 AgrD), purple (H77 AgrB-II), fuchsia (E34 AgrD), green (D33 AgrD), yellow (K139 AgrB-I) and orange (R70 AgrB-I). All AgrB termini are on the cytosolic side (top). The (AgrB)_2_/AgrD complex orientation here is shown following the convention of Figure 2 with cytosol at the top offering a better view of the cytosol-accessible active site. Tilt in AgrB helices 2 and 5 (orange-to-cyan), induced by peripheral binding of AgrD to AgrB from the cytosol is shown in **(B)**. Pairing between AgrB-K139 in H5 and AgrD-D29 pulls the top of H5 while hydrophobic interaction tilts H2 (orange-to-cyan). Tilt in H5 in membrane AgrB_2_/AgrD complex **(C)** driven by AgrB-K139/AgrD-D33 contact (*cf.* Panel **(A)**.

Structural data is lacking for any orthologs in the AgrB family of transmembrane endopeptidases, or for the AgrD pro-peptide. To understand the molecular conformations and relate these to the molecular interactions occurring between AgrB and AgrD, we established MD-annealed homology models. We obtained a homology model of AgrB using LOMETS (Wu and Zhang, 2007), a component of the I-TASSER suite of programmes (Zhang, 2008). This model revealed six transmembrane domain helical topology (MD-annealed structure in Figure 2A), which is also reflected in secondary structure prediction from JPred 4 server (Drozdetskiy et al., 2015) and supported by the CD data (Figure 6). The LOMETS model of AgrB shows both N- and C- termini on the same side and places amino acid residues known to affect function on the *cis* side near the polar/apolar interface. These include the active site cysteine, C84, responsible for cleaving AgrD1 between M32 and D33 (Figure 1), which is located in the membrane interior providing access to the hydrophobic signalling pro-peptide substrate (Figure 3A). Other AgrB residues near the active site and required for proteolytic activity include R70, G75 and H77 (Qiu et al., 2005; Thoendel and Horswill, 2013) which reside on the cytoplasmic side at the end of helix 2 and in a tighter turn towards helix 3 (Figure 3A). Mutations in K129-131 have been reported to retain peptidase activity but not AIP formation (Thoendel and Horswill, 2013). These are located on a cytoplasmic loop in AgrB-I, which remains mobile but can interact with C-terminal E186 and D187 on AgrB-II to stabilise the (AgrB)_2_ dimer complex. The loop containing K129-131 stems from tilted helix 5, observed in AgrB-I associated with AgrD (Figure 3A and C).

The AIP pro-peptide AgrD1 was modelled using I-TASSER and the best model was a tight, symmetrical alpha helical hairpin. After annealing in aqueous solution, the peptide with high conformational plasticity underwent significant rearrangement to reveal overall helical fold with flexible segments and an intrinsically disordered overall fold. Such flexibility is important for enabling conformational adaptation of the peptide to varied physicochemical environments from aqueous, through membrane-associated to inserted, AgrB-associated states.

### Molecular complex assembly of AgrB and AgrD–Conformational analysis and MD simulations

To remove residual template-inherited conformational strain from the modelling, we annealed the AgrB and AgrD models separately in simulated lipid membrane patches by all-atom molecular dynamics simulations. Proteolipid systems were assembled and hydrated using CHARMM-GUI (Jo et al., 2008) and all-atom simulations were carried out using NAMD (Phillips et al., 2005). MD trajectories were calculated for 500 ns for AgrB and AgrD, each independently embedded in membrane patches of POPE/POPG/CL (75:20:5). The AgrB structure showed very little template-inherited conformational strain and annealed within 30 ns to RMSD variation on the order of 4 Å with little deviation from the original model (Supplementary Figure S1A).

The AgrD structure, by contrast, evolved significantly until 200 ns and reached equilibrium only during the last 100 ns of the evolution trajectory. The final AgrD structure differed from the original tight helical hairpin model and became more oblate in shape with curved helices within the membrane approaching a toroidal shape with axis inclined at 45° to the What is a membrane normal Towards the end of the simulation the equilibrated pro-peptide structure presented a hydrophilic face that led to the formation of a trans-membrane water channel across the membrane, seen as water ingress into the membrane interior (shown as presence of water oxygens (Figure 2B, red), alongside the peptide between the phosphate (cyan) planes of the membrane (*cf.* Figure 2B). The formation of such a water channel is corroborated by experimental observations of membrane conductance in the presence of AgrD (Schwartz et al., 2014). The annealed conformations of AgrB and AgrD at the end of 500 ns of atomistic simulations in a membrane are shown in Figure 2A,B, respectively. By contrast, the AgrD conformation in solution adjusted rapidly and molecular RMSD remained levelled after the first 20 ns, indicating significantly slower conformational dynamics of the peptide in membranes compared to solution.

We are seeking a mechanistic understanding of the interaction between the endopeptidase AgrB and its substrate, the pro-peptide AgrD at the atomic level of detail. Since atomistic simulation of the entire process is not practically achievable for spontaneous insertion of AgrD, the individual stages of the process were modelled on the bases of known key steps. Extending the simulations to 1.0 without AgrB and to 1.5 μs with AgrB (monomer or dimer) did not lead to AgrD insertion, which may indicate a role of MroQ alone or in combination with AgrB as facilitator of the membrane insertion of AgrD. The involvement of MroQ in AIP pre-processing has recently been highlighted (Zhao et al., 2022).

We began with AgrD equilibrated in solution, which conformationally can be described as containing helical segments within an overall flexible, structure (*cf.* RMSD in Supplementary Figure S1A). This conformation was presented at 30 Å above a lipid membrane surface (Supplementary Figure S1B) and after 500 ns of unbiased MD simulations, AgrD associated via its helical N-terminal domain with the membrane surface with its C-terminus remaining largely flexible and solvated (Figure 2C). The same docking process was repeated in the presence of AgrB in the membrane (Figure 2D). AgrD associated with AgrB *via* its N-terminal short helix, which engaged helix 5 of AgrB and subsequently drove a significant tilt in helix 2 mediated by H2/N-AgrD hydrophobic contact (Figure 2D). This contact was led by D29 and D33 of AgrD near substrate C28 (green), which interacted electrostatically with R136 and K131, respectively, located on a flexible loop on the cytosolic side of AgrB between H4 and H5 (Figure 2D). Surface associated AgrD displaced lipid headgroups to engage and significantly tilt AgrB H2 (Figure 3B).

Motional restrictions within the bilayer significantly reduce molecular mobility and lateral MD docking is unrealistic due to prohibitively long simulation times. To elucidate the next stage of interaction, models of AgrD/AgrB, as well as of AgrD/AgrB_2_ dimer were obtained using rigid docking from equilibrated structures and embedded in membranes for 500 ns atomistic MD simulations. The annealed conformation of AgrB-I/AgrD reveals a stable complex, in which C28-C84 proximity is maintained and a tilt in AgrB-H5 was observed (Figure 3C). Membrane-embedded AgrD complexes with AgrB and AgrB_2_ both lead to local deformation of the bilayer and lipid headgroup ingress into the membrane forming a lipid-lined aqueous channel (Figure 2D,F).

The ternary (AgrB)_2_AgrD complex, following 500 ns annealing, reveals non-equivalent positions of the two AgrB monomers, AgrB-I and AgrB-II. AgrB-I appears to resemble conformationally AgrB in its complex with surface-associated AgrD (Figure 3B), while monomer AgrB-II resembles the conformation of membrane-embedded complex (Figure 3C). This puts forward the mechanistic model, in which AgrB-I is responsible for pro-peptide insertion and docking in the correct orientation for catalytic processing by AgrB-II (Figure 3A). AgrD is positioned with AgrD C28 in proximity to C84 of AgrB-II and stabilised in place via AgrB-I-K139/AgrD-D33 and by AgrB-II-R70/AgrD-E34 (Figure 3A). AgrD-induced tilt in H5 of AgrB-I and in H3 of AgrB-II is conformationally similar to AgrB in complexes with surface-associated and with membrane-embedded AgrD, respectively.

### Molecular interactions between AgrB-AgrB and AgrB-AgrD *in vivo*

**FIGURE 4:**
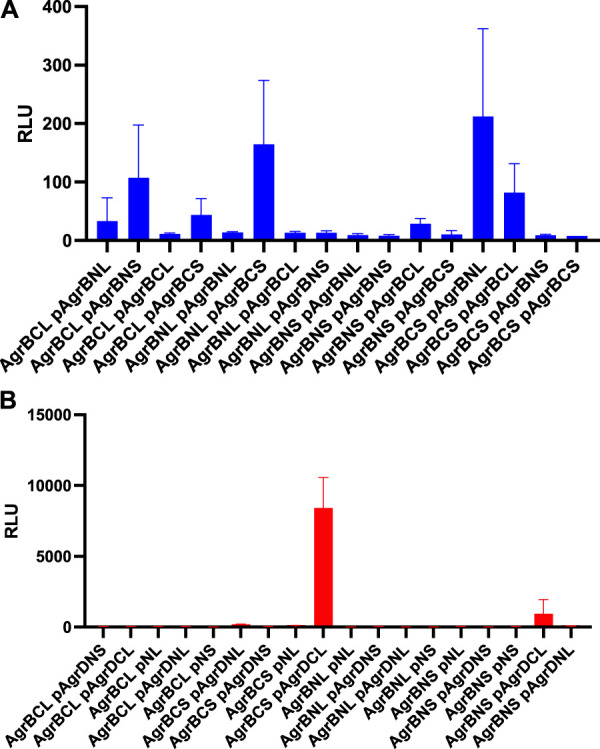
AgrB interacts with itself and with AgrD *in vivo* in *Staphylococcus aureus*. AgrB-AgrB and AgrB-AgrD protein-protein interactions in cells were explored using split luciferase assays. AgrB and AgrD were tagged with either N-terminal LgBiT, N-terminal SmBiT, C-terminal LgBiT or C-terminal SmBiT. The corresponding genetic constructs were used to make a combinatorial series of *Staphylococcus aureus* strains by introducing a tagged *agrB* gene onto the chromosome and a second tagged *agrB* or tagged AgrD on a plasmid (p). On the *x*-axis labels, N, N-terminus, C, C-terminus, S, SmBiT, L, LgBiT. For some experiments, plasmid controls expressing the LgBiT (pNL) or SmBiT (pNS) only were included. Reconstitution of the luciferase was assayed by quantification of bioluminescence (RLU) following the addition of furimazine. The strains used are listed in Supplementary Table S3. **(A)** High light output indicating strong AgrB-AgrB interactions were observed only when the N- and C- terminally tagged AgrBs were both present. **(B)** High affinity AgrB-AgrD protein-protein interactions were almost exclusively observed when C-terminally tagged AgrB and AgrD were present as AgrB-SmBiT and AgrD-LgBit (see AgrBCS and pAgrDCL). No bioluminescence (RLU) was observed for the plasmid controls (pNL and pNS) in the presence of C-terminally tagged AgrB with either luciferase sub-unit. The strains used are listed in Supplementary Table S4. Data are representative of three technical repeats recorded for each sample with error bars displaying standard deviation. Experiments were carried out in biological triplicate.

To determine whether AgrB-AgrB and AgrB-AgrD intramembrane protein-protein interactions could be demonstrated experimentally in cells, we used NanoLuc^®^ Binary Technology (NanoBiT). This employs the LargeBit (LgBiT; 114 amino acids) and the complementary SmallBit (SmBiT; 11 amino acids) optimized to have a low affinity for the LgBit. When LgBit and SmBiT expressed as fusions to target proteins interact, they form an active luciferase. To explore AgrB interactions with itself in *S. aureus* cells, we fused the LgBiT or SmBiT to the N- or the C- termini of AgrB or *vice versa* and introduced the fusions onto an ectopic site on the chromosome or onto a plasmid in the same cell, both driven by the *agr* P2 promoter. Similarly, as controls we cloned the LgBiT and SmBit alone into the same sites. If dimerization of AgrB occurs then the active luciferase will be reconstituted and detectable in live cells as bioluminescence upon provision of furimazine. Figure 4 shows that the generation of bioluminescence indicative of AgrB interacting with itself were primarily observed for the constructs in which the N- and C- termini tagged respectively with the LgBiT and SmBiT or *vice versa* were present. A weaker interaction was observed for the AgrB C-C termini. These data also suggest that AgrB must form at least a dimer, in which the AgrB N- and C- termini and possibly the C-C termini on different AgrB monomers are sufficiently close to reconstitute the luciferase. This contrasts with the N-termini that do not appear to interact in this assay.

Using the same split-luciferase strategy, we also tagged AgrD with the SmBiT or LgBiT or *vice versa* at the N- and C- termini. Figure 4B shows a very strong luminescent signal >10 times that of the other constructs for the C-terminal fusions of AgrB and AgrD with the Smbit and LgBiT respectively indicative of a high affinity interaction between AgrB and the C-terminus of AgrD. This is consistent with the primary function of AgrB in cleaving the 14 AgrD C-terminal amino acids and driving thiolactone formation (Figure 1).

### AgrBD interactions in vitro

**FIGURE 5:**
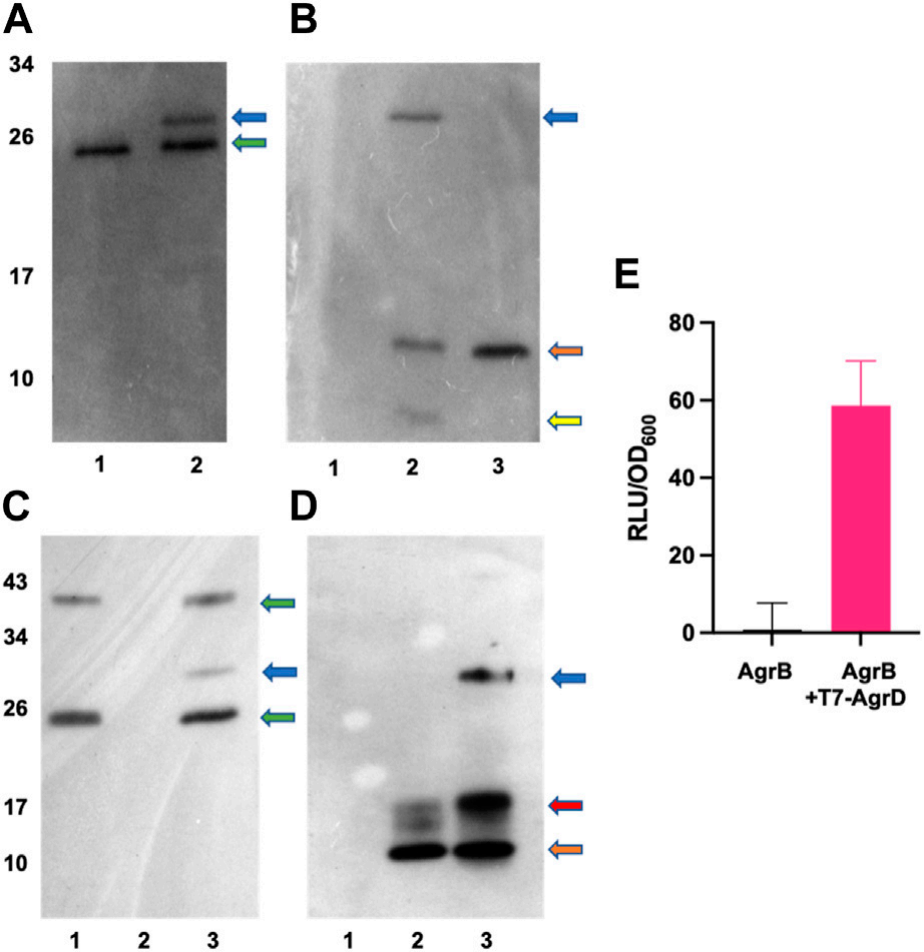
*In vitro* AgrBD complex formation and AIP production via (E) *coli* membrane preparations or purified AgrB and synthetic T7-AgrD. **(A)** Western blot of membranes prepared from *Escherichia coli* expressing AgrB without (lane 1) or with (lane 2) T7-AgrD and probed with a nanobody to AgrB. **(B)** As **(A)** but probed with an antibody to T7-AgrD and where lane 1 (AgrB), lane 2 (AgrB + T7-AgrD) and lane 3 (T7-AgrD). In **(C)** and **(D)** the lanes are lane 1 (AgrB), lane 2 (T7-AgrD) and lane 3 (AgrB + T7-AgrD) but with purified AgrB protein and DOPG instead of *Escherichia coli* membranes. The blots shown in **(C)** and **(D)** were probed with the nanobody to AgrB and antibody to T7-AgrD respectively as described in the methods. The positions of the AgrBD complexes in **(A–D)** are highlighted with a blue arrow, the green arrows indicate the AgrB monomers and dimers, the orange arrows indicate the position of T7-AgrD, the red arrow in **D**, the position of an AgrD-T7 dimer and the yellow arrow in **B**, C-terminally processed T7-AgrD. **(E)** Membranes from *Escherichia coli* expressing AgrB incubated with or without T7-AgrD and assayed for AIP production using the AIP-1 biosensor ROJ143.

To investigate experimentally the interaction between AgrB and AgrD *in vitro*, we initially used membranes prepared from *E. coli* expressing AgrB or as a control, membranes from *E. coli* transformed with the empty vector pCDFDuet-1 The membranes were incubated with or without a synthetic T7-tagged AgrD peptide. Supplementary Figure S4 shows that T7-AgrD is not processed by membranes prepared from the *E. coli* control strain and that T7-AgrD itself does not activate the AIP-1 reporter strain ROJ143. On Western blots the formation of a new protein band at ∼30 kDa (Figure 5A, lane 2 and Figure 5B lane 2) was observed after 30 min incubation. Since this band migrated at the combined mass of the AgrB monomer and T7-AgrD and reacted with both the AgrB nanobody (Figure 5A, lane 2) and the T7 tag monoclonal (Figure 5B, lane 2), we can conclude that it is the AgrBD complex. Samples taken from this assay activated the ROJ143 reporter strain and therefore contain AIP-1 (Figure 5E) as anticipated since membranes prepared from *E. coli* expressing *agrB1* are capable of removing the N-terminal amphipathic leader from AgrD after AgrB-dependent cleavage of the AgrD C-terminus and formation of the thiolactone macrocycle (Thoendel and Horswill, 2009; Thoendel and Horswill, 2013). The *E. coli* membrane protease(s) involved has not yet been identified.

To confirm AgrBD complex formation and hence the functionality of AgrB after purification, the recombinant protein was expressed and purified (Supplementary Figure S3) as described in the Methods. Figure 5C (lane 3) and Figure 5D (lane 3) show the AgrBD complex formed when AgrB was incubated with T7-AgrD in the presence of a charged phospholipid (DOPG) which we found to be essential for AgrB activity. Wang et al. (2015a) have provided experimental evidence that phospholipids stabilize the N-AgrD thiolactone formed during proteolytic cleavage of AgrD by AgrB.

### Molecular complexes and thermal malleability of AgrB-AgrD monitored by SRCD

**FIGURE 6:**
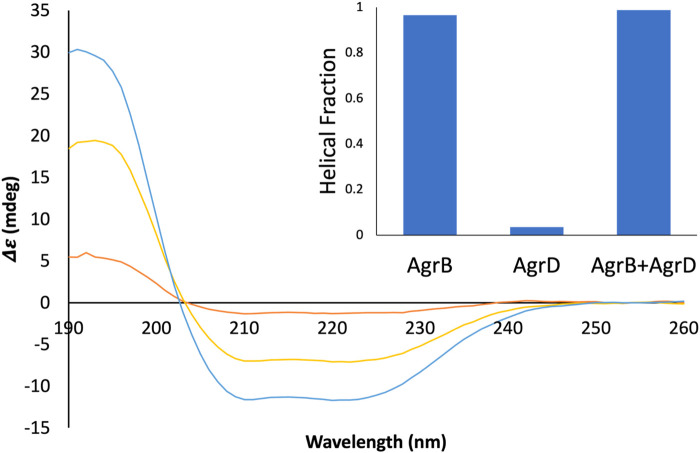
SRCD spectra from AgrB, AgrB_2_/AgrD and AgrD and corresponding helical content. Δε spectra from 0.13 mg/mL AgrB (yellow), 0.13 mg/mL AgrB/AgrD 1:1 molar ratio (blue) and 1.0 mg/mL AgrD (red) in DDM; and corresponding secondary structure content (inset). Higher concentration of AgrD was needed to estimate the helical fraction in (B) due to the very weak signal from AgrD at 0.13 mg/mL. Despite the negligible direct contribution from AgrD to helicity from the AgrB/AgrD complex, the observed helical fraction is higher than in AgrB alone. The SRCD spectra from AgrB, AgrD and stoichiometric AgrB/AgrD mixtures at 0.13 mg/mL are shown in Supplementary Figure S2.

To confirm molecular complex formation between AgrB and AgrD *in vitro*, we monitored temperature-induced changes in protein secondary structure by SRCD, which offers quantitative insights into secondary structure in response to molecular interactions (Greenfield, 2006; Hussain et al., 2018). Recombinant AgrB was reconstituted into membrane mimicking DDM proteodetergent micelles, which maintain its folded conformation in optically transparent suspensions. The SRCD studies revealed almost 100% helical content from AgrB alone, which increased to 100% in the presence of substrate AgrD (Figure 6). The high helical content combined with AgrB sequence length, corroborates the predicted 6TMD topology. AgrB secondary structure content remains almost unchanged in the presence of AgrD, which confirms the proper fold is retained and we employ thermal analysis to quantify the molecular interaction between the endopeptidase and its substrate.

**FIGURE 7:**
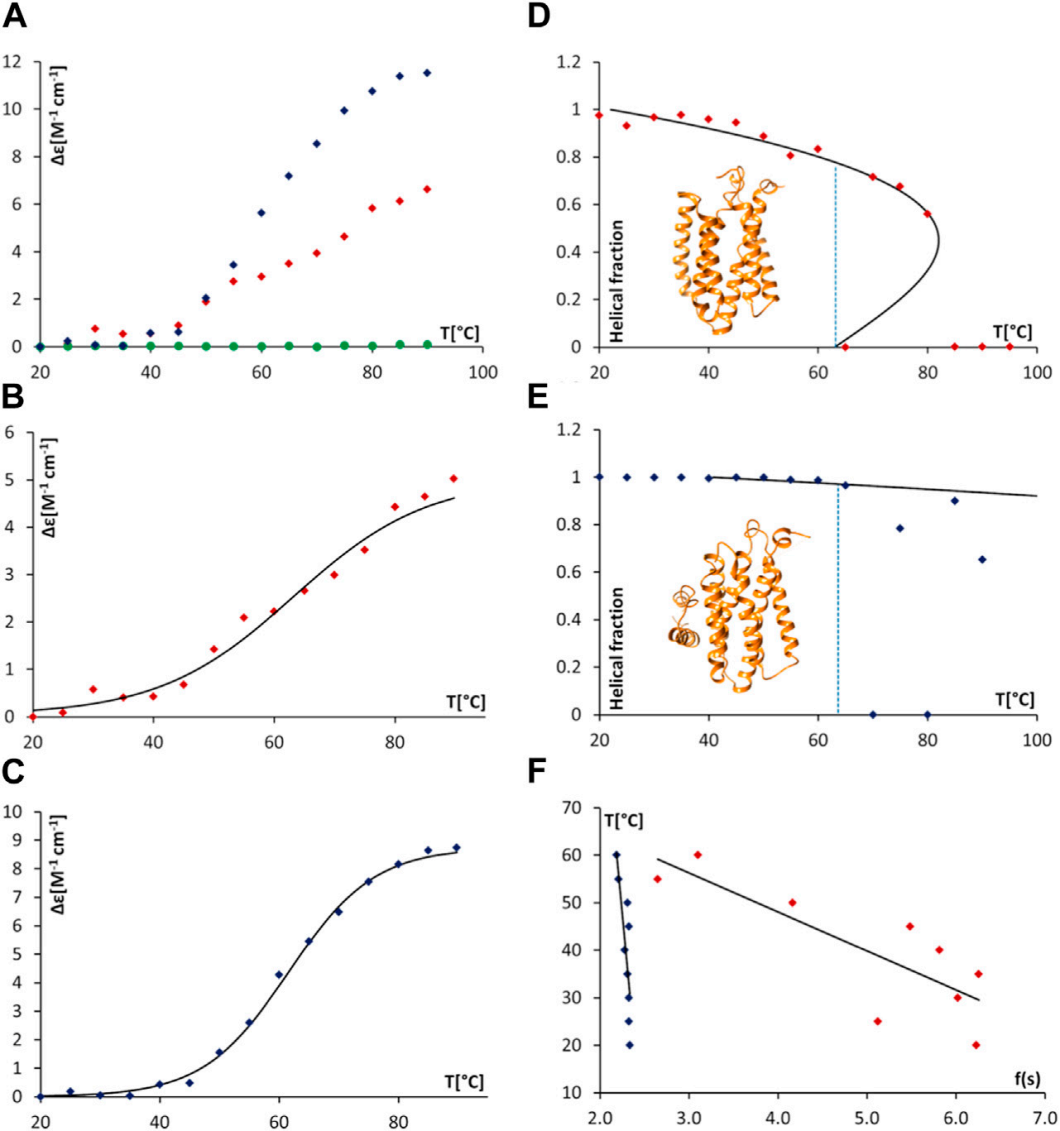
Thermal unfolding of proteodetergent AgrB and AgrB_2_/AgrD micelles monitored by SRCD and spinodal analysis using Landau free energy model. Changes Δε(210) versus temperature **(A)** for AgrB (red diamonds), AgrD (green circles) and AgrB + AgrD (blue diamonds) in DDM micelles; Δε(210) of AgrB with a sigmoidal fit **(B)** *T*_*m*_ = 63°C; and, Δε(210) of AgrB + AgrD in DDM micelles with a sigmoidal fit, *T*_*m*_ = 62°C **(C)**. Helical fraction as a function of temperature for AgrB **(D)** and AgrB + AgrD **(E)** with an equation of state obtained from a Landau free energy expansion model (Hussain et al., 2018); insets in **(D)** and **(E)**—models of AgrB and of AgrB/AgrD, respectively; and, **(F)** linear fits of *T* vs. $f\left(s\right)={3\left(\frac{s-s_{+}}{s_{+}}\right)}^{2}+{\left(\frac{s-s_{+}}{s_{+}}\right)}^{3}$ , used to calculate the equations of state in **(D)** and **(E)** where *s* is the helical order parameter and *s*_*+*_ is the positive spinodal (Hussain et al., 2018). The corresponding spinodal parameters for AgrB are *T*_*+*_ = 81°C, *s*_*+*_ = 0.44 (*τ*_*+*_ = 0.054) and for AgrB + AgrD: *T*_*+*_ = 209°C, *s*_*+*_ = 0.56 and *T*_*+*_ = 209°C (*τ*_*+*_ = 0.43).

Thermal denaturation assays, monitored by SRCD were then undertaken to obtain complementary data in support of direct AgrB/AgrD interactions presented above using cellular and biochemical assays. The thermal unfolding transition of the largely helical AgrB was estimated by following Δε intensity change at 210 nm as a function of temperature and revealed the unfolding transition occurring at 63°C. The transition appeared more cooperative in the presence of AgrD, judged by the slightly narrower temperature interval around the inflection (Figure 7A,B,E and F). The CD spectrum of AgrD in DDM mixed micelles is tenfold lower, does not provide a distinct melting point (Figures 7C,D) and so was excluded from subsequent analysis (Supplementary Figure S2).

To obtain a quantitative measure of the impact of substrate binding on protein stability we analysed the thermal unfolding SRCD data from AgrB alone and with AgrD using a Landau free energy expansion model (Hussain et al., 2018). Changes in α-helical content of AgrB in DDM were followed as a function of temperature without (Figure 7A) and with the AgrD substrate (Figure 7E). An equation of state for the thermal response of AgrB was obtained and fitted to the data for the folded protein alone without comparison to secondary structure content in the unfolded, denatured state.

**FIGURE 8:**
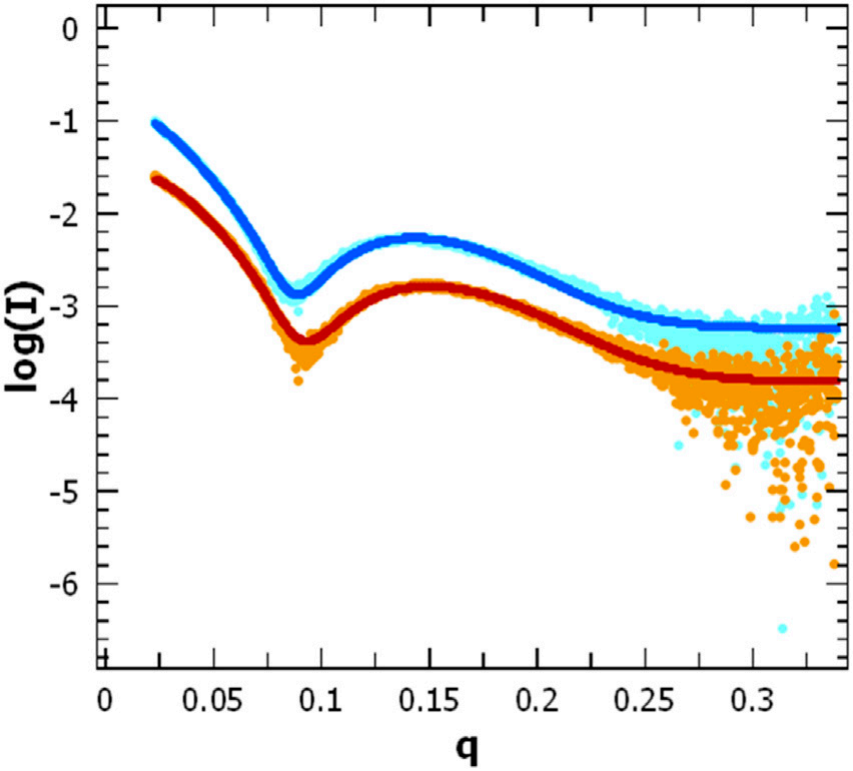
Scattering pattern of AgrB in DDM proteomicelles. Intensity log plots vs. q-factor for AgrB alone (light blue) and corresponding data fitting (dark blue) are compared to the AgrB/AgrD complex scattering (orange) and data fitting (red). The fits were obtained using SasView 5.0.5 and visualised using PRIMUS. The data for the fit were restricted to a q-range starting from 0.0237 A^-1^. The data and corresponding fits are scaled for clarity. (right).

We quantify transition cooperativity, related to the first order “strength” of the unfolding transition, as reflected in the reduced spinodal temperature *τ*_*+*_ = 1—*T*_*+*_/*T*_*m*_, which includes both thermal width of the transition and dependence on the change in magnitude of spinodal order *Δs* = *s*_*+*_—*s*_*-*_ = *s*_*+*_/2. A unique and major advantage of using *τ*_*+*_ as a measure of transition strength is its relative insensitivity T_m_, which results from dependence of *T*_*+*_ on *T*_*m*_ that is only determined by the fit to experimental data. The helical order data from Figure 8 can be replotted in a linear form using the quantity f(s) (*cf.* Methods section). Linear fits were then used to obtain the spinodal parameters from the melts of AgrB (Figure 7B), AgrB/AgrD (Figure 7D) and both together Figure 7F) and to quantify substrate-induced changes in AgrB thermal stability.

Protein secondary structure is unaffected by temperature below 30°C and because the series expansion is defined near the unfolding transition, we excluded from analysis data below 30°C and above *T*_*m*_. This did not affect the comparison and had little effect on the location of the spinodals but provided a more accurate equation of state near the transition. The presence of AgrD makes the helical content of AgrB insensitive to temperatures close to *T*_*m*_ and we considered only the range between 50°C and 65°C, where some changes in helicity were observed. The fitting yielded the spinodal point of AgrB alone at *T*_*+*_ = 81°C and *s*_*+*_ = 0.44, while in the presence of AgrD the spinodal shifts to *T*_*+*_ = 209°C and *s*_*+*_ = 0.56. These parameters were then used to calculate the equation of state, shown alongside the thermal melts of AgrB alone and with AgrD in Figures 7B,F respectively.

This analysis revealed a well-behaved monotonic melt of AgrB and significant increase in resistance to melting in the presence of AgrD, which proceeds with a high cooperativity transition. Examination of the α-helical content in AgrB above *T*_*m*_ shows that the α-helical content continues to follow the predicted equation of state as far at *T*_*+*_, revealing that even above *T*_*m*_ the α-helical content and, likely the fold, are maintained in a metastable state. Above *T*_*+*_ AgrB shows no residual α-helical content and reaches an irreversibly denatured state, from which temperature reduction does not recover the original fold.

Significant elevation in *T*_*+*_ from 81°C for AgrB in DDM to 209°C in the presence of AgrD, reflected in transition cooperativity change from *τ*_*+*_ = 0.054 to *τ*_*+*_ = 0.43, shows enhanced structural stability of AgrB in the presence of AgrD and marked resistance to thermal denaturation in the presence of substrate. The melt is highly cooperative, and a total structural collapse occurs almost immediately above *T*_*m*_. The presence of AgrD has a strong stabilising effect on AgrB up to *T*_*m*_, above which secondary structure oscillated between 0 and some degree of folding, which is below the equation of state. This observation provides strong evidence of existence of tight molecular complexes between AgrB and AgrD *in vitro*, in which large molecular surface on AgrB is interfaced and supported by AgrD, while unchanged transition temperature *T*_*m*_ suggests that the main driver of AgrB unfolding is the bonding energy, rather than number of bonds responsible for stabilising protein structure. The conformational plasticity observed in AgrB permits TM domain excursions illustrated in the MD evolution trajectories (Figure 2G), for example, H5 tilt, associated with AgrD binding. The final (AgrB)_2_/AgrD complex, by contrast, is stabilised by additional intermolecular bonding networks and lacks such conformational adaptability and thermal malleability (Figure 2K,L).

### Impact of AgrD on AgrB structure and oligomerization as determined by SAXS

To assess changes in the overall size and shape of AgrB proteodetergent micelles on association of AgrD, we investigated the system using solution SAXS. Scattering patterns obtained from AgrB or AgrB/AgrD in DDM proteomicelles were analysed using SasView 5.0.5 (Doucet et al., 2021) and showed that best fit was obtained using a core-shell ellipsoid model (Kotlarchyk and Chen, 1983; Berr, 1987) (Figure 8).

The core dimensions associated with (AgrB)_2_ dimer, derived from fitting the SAXS experiment, did not differ significantly without or with AgrD, which suggests that AgrD binding does not affect the oligomerisation state of AgrB (Figure 8). The shell thickness, however, appears reduced in the presence of AgrD, which reveals partial deconstruction in the micellar belt. This is seen as a shift in the thickness of the shell from 24.0 ± 0.1 to 22.3 ± 0.1 Å and corroborates the formation of an aqueous channel near AgrD that disrupts the detergent micelle, as observed in the MD simulations (Figure 2M) but does not occur in the absence of AgrD. At the same time, the core radius slightly decreases from 10.60 ± 0.05 to 10.51 ± 0.04 Å, in agreement with increased AgrB structural stability observed in thermal melts by CD (Figure 7). Other parameters such as the core axial ratio and the ratio of thickness of the shell at pole to that at equator are also reported, varying from 8.0 ± 0.2 to 5.5 ± 0.1 and 0.41 ± 0.05 to 0.54 ± 0.03 respectively, upon addition of AgrD. This supports the rearrangement of the micelle described above.

## Conclusion

Membrane proteins are important, yet challenging targets, which are present in low molarity in cells and require hydrophobic support of their native fold for *in vitro* molecular and structural analysis. The staphylococcal quorum sensing membrane endopeptidase, AgrB, responsible for processing AgrD, is conserved in most Gram-positive bacteria including a number of important pathogens (e.g., staphylococci, enterococci, clostridia, and *Listeria*) and is considered a potential target for novel anti-infectives (Nakayama et al., 2009; Gordon et al., 2013; Murray et al., 2014).

In this study, computational, biophysical, biochemical and cellular methodology were used to demonstrate molecular complex formation between AgrB and AgrD *in vitro* and *in vivo*, and to characterise the mechanisms of self-assembly, molecular organisation and stability of these membrane protein complexes. An alpha helical structural model of AgrB with 6TMD topology is proposed and corroborated with helical predictions and helical content from CD. The functionality and ability of recombinant AgrB to process AgrD was confirmed and the formation of molecular complexes between AgrB and AgrD *in vitro* and *in vivo* validated.

The AgrD-AgrB interaction can be visualized via distinct steps, beginning with AgrD interacting from the aqueous cytosol with lipid membranes and with membrane-embedded AgrB, followed by a conformational adaptation of AgrB in the complex. The formation of AgrB dimers *in vivo* using a split luciferase assay and use of atomistic MD simulations to investigate the structure and stability of ternary (AgrB)_2_AgrD membrane complexes are shown. We propose a model with two non-equivalent AgrB sites, in which one monomer (AgrB-I) facilitates insertion and positioning of AgrD in the correct orientation for catalytic processing by the second, AgrB-II monomer (Figure 3). The MD evolution trajectories lead to stable molecular assemblies, in which we propose the role for key residues R70 and K139 from AgrB-I, and D33 and E34 from AgrD as mediators of complex stability and in positioning C84 against C28 and M32 in catalytic proximity (Figure 3). AgrB with a R70G substitution is known to lack peptidase activity (Thoendel and Horswill, 2013), while D33 and E34 in the AgrD C-terminal tail were essential for cleavage by AgrB (Thoendel and Horswill, 2009). In our model, all of these residues take strategic positions in stabilising AgrD within a non-equivalent AgrB dimer via charge-charge interactions, aided by π-interactions between H77 from AgrB-2 and F30 from AgrD. However, further work will be required to experimentally confirm the (AgrB)_2_AgrD complex structure and whether the higher order AgrB multimers apparent on polyacrylamide gel electrophoresis (Supplementary Figure S3; Wang et al., 2015b) also form *in vivo*. Whether the AgrB monomer alone possesses enzymatic activity also remains to be established.

Using a novel biophysical framework, based on SRCD-monitored structural plasticity of membrane proteins, the significant conformational malleability of the AgrB structure that permits the proposed conformational rearrangements in accommodating incoming substrate, AgrD was apparent. The mature complex shows significant structural stability and lacks such plasticity almost entirely. Throughout the stages of molecular interaction with membranes and AgrB, the substrate AgrD retains high structural flexibility that allows it to adapt to environments starting from aqueous solution through membrane associated to membrane integrated and AgrB-bound states. Membrane insertion and AIP processing is facilitated by the formation of a lipid-lined aqueous channel alongside the AgrB_2_-AgrD complex.

## Data Availability

Publicly available datasets were analyzed in this study. This data can be found here: https://www.uniprot.org/uniprotkb/P0C1P7/entry. All data needed to evaluate the conclusions in the paper are present in the paper and/or the Supplementary Materials.
