# Supplementary material for: Conformational analysis and interaction of the Staphylococcus aureus transmembrane peptidase AgrB with its AgrD propeptide substrate
Source: Front Chem. 2023 May 5;11:1113885. doi: 10.3389/fchem.2023.1113885 (PMC10196373; doi:10.3389/fchem.2023.1113885)
Supplement: Supplementary file 1 [file DataSheet1.docx]

**Supplementary Information**

**Conformational analysis and interaction of the *Staphylococcus aureus* transmembrane peptidase AgrB with its AgrD propeptide substrate**

*Philip Bardelang^1^, Ewan Murray^1^, Isobel Blower^1,2^, Sara Zandomeneghi^1^, Alice Goode^1^, Rohanah Hussain^3^, Divya Kumari^1^, Giuliano Siligardi^3^, Katsuaki Inoue^3^, Jeni Luckett^1^, James Doutch^4^, Jonas Emsley^5^, Weng C. Chan^5^, Philip Hill^2^, Paul Williams^1*‡^ and Boyan B. Bonev^1*‡^*

^1^Biodiscovery Institute and School of Life Sciences, University of Nottingham, Nottingham NG7 2UH, U.K.

^2^School of Biosciences, University of Nottingham, Sutton Bonington Campus, University of Nottingham, Loughborough LE12 5RD, UK

^3^Diamond Light Source Ltd., Harwell Science and Innovation Campus, Chilton, Didcot, Oxfordshire, OX11 0DE, UK

^4^ISIS Neutron and Muon Source, Rutherford Appleton Laboratory, Harwell Oxford, Didcot OX11 0QX, UK;

^5^School of Pharmacy, Biodiscovery Institute, University of Nottingham, University Park, Nottingham NG7 2RD, UK

^‡^Joint senior authors

^*^ Correspondence: [boyan.bonev@nottingham.ac.uk](mailto:boyan.bonev@nottingham.ac.uk); tel. +44 115 823 0177; [paul.williams@nottingham.ac.uk](mailto:paul.williams@nottingham.ac.uk); tel. +44 115 951 5047

**Table S1**. Bacterial Strains and Plasmids

| **Strains/Plasmids** | **Relevant Characteristics** | **Reference/Source** |
| --- | --- | --- |
| ***E. coli*** |  |  |
| DC10B | Cloning host; *dcm*^-^ Str^R^ | Monk *et al* (2012) |
| C41 (DE3) | BL21 derivative host for recombinant membrane protein expression | Miroux & Walker (1996) |
| C43 (DE3) | BL21 derivative host for recombinant membrane protein expression for Spc^R^ | Miroux & Walker (1996) |
| ***S. aureus*** |  |  |
| USA300 | CA-MRSA strain; agr-I | McDougal *et al* (2003) |
| CYL12349 | RN4220 *attB2* pYL112Δ19 | Lei *et al* (2012) |
| ROJ143 | ROJ48 pAgrP2C1A; AIP bioreporter | Jensen *et al* (2008) |
| SH1000 | Functional *rsbU* derivative of 8325-4, *rsbU*^+^ | Horsburgh *et al* (2002) |
| SH1000 attB2:: SK-P2-*agrB* | *agrB* inserted into chromosomal *attB2* site | This laboratory |
| SH1000 attB2:: SK-P2-NL*agrB* | N-terminal LgBiT-*agrB* fusion into chromosomal *attB2* site | This laboratory |
| SH1000 attB2:: SK-P2-NS*agrB* | N-terminal SmBiT-*agrB* fusion into chromosomal *attB2* site | This laboratory |
| SH1000 attB2:: SK-P2-*agrB*CL | C-terminal LgBiT-*agrB* fusion into chromosomal *attB2* site | This laboratory |
| SH1000 attB2:: SK-P2-*agrB*CS | C-terminal SmBiT-*agrB* fusion into chromosomal *attB2* site | This laboratory |
| **Plasmids** |  |  |
| pCDFDuet-1 | T7 promoter; gene expression vector; Sm^R^ | Novagen |
| pCDFDuet-*agrB1* | *agrB1* expression vector | This laboratory |
| pCDF-Duet-NH-*agrB1* | *agrB1-Hibit* expression vector |  |
| pCDF-Duet-*agrB1*-CH | *Hibit-agrB* expression vector |  |
| pSK5630 | pSK1 Amp^R^ Cm^R^ | Grkovic *et al* (2003) |
| pSK-P2 | pSK5630 with *agr*P2 promoter | This laboratory |
| pSK-P2-*agrB* | pSK-P2 with *agrB* expressed from *agr*P2 promoter | This laboratory |
| pSK-P2-NS | pSK-P2 expressing N-terminal SmBiT | This laboratory |
| pSK-B2-NL | pSK-P2 expressing N-terminal LgBiT | This laboratory |
| pSK-P2-NL*agrB* | pSK-P2 with N-terminal LgBiT fusion to AgrB | This laboratory |
| pSK-P2-NS*agrB* | pSK-P2 with N-terminal SmBiT fusion to AgrB | This laboratory |
| pSK-P2-*agrB*CL | pSK-P2 with C-terminal LgBit fusion to AgrB | This laboratory |
| pSK-P2-*agrB*CS | pSK-P2 with C-terminal SmBiT fusion to AgrB | This laboratory |
| pLL102k | pLL102 Km^R^ | This laboratory |
| pSK-P2-NL*agrD* | pSK-P2 with N-terminal LgBiT fusion to AgrD1 | This laboratory |
| pSK-P2-NS*agrD* | pSK-P2 with N-terminal SmBiT fusion to AgrB1 | This laboratory |
| pSK-P2-*agrD*CL | pSK-P2 with C-terminal LgBiT fusion to AgrD1 | This laboratory |
| pDG729 | Km^R^ | Guerout-Fleury *et al* (1995) |
| pLL102 | Derivative of pCL25 carrying *attP2* TetR | Lei et al, (2012) |
| pLL102k | pLL102 Km^R^ | This laboratory |
| pAgrP2C1A | pSKerm *agr*P2 *agrC1 agrA* CmR | Jensen *et al* (2008) |
| pCOLD1 | Cold shock expression vector; Amp^R^ | Takara |
| pCOLD1-*agrB1* | *agrB1* expression vector; Amp^R^ | This study |
| pRARE2 | Carries seven rare-codon tRNA genes for overcoming codon bias; Cm^R^ | Novagene |

**References**

Grkovic S, Brown MH, Hardie KM, Firth N, Skurray RA. Stable low-copy-number *Staphylococcus aureus* shuttle vectors. *Microbiology*. 2003;149:785-794.

Guérout-Fleury AM, Shazand K, Frandsen N, Stragier P. Antibiotic-resistance cassettes for Bacillus subtilis. *Gene*. 1995;167:335-336.

Jensen RO, Winzer K, Clarke SR, Chan WC, Williams P. Differential recognition of *Staphylococcus aureus* quorum-sensing signals depends on both extracellular loops 1 and 2 of the transmembrane sensor AgrC. *J Mol Biol*. 2008; 381:300-309.

Horsburgh MJ, Aish JL, White IJ, Shaw L, Lithgow JK, Foster SJ. sigmaB modulates virulence determinant expression and stress resistance: characterization of a functional *rsbU* strain derived from *Staphylococcus aureus* 8325-4. *J Bacteriol*. 2002;184(19):5457-5467.

Lei MG, Cue D, Alba J, Junecko J, Graham JW, Lee CY. A single copy integration vector that integrates at an engineered site on the Staphylococcus aureus chromosome. *BMC Res Notes*. 2012; 5:5.

McDougal LK, Steward CD, Killgore GE, Chaitram JM, McAllister SK, Tenover FC. Pulsed-field gel electrophoresis typing of oxacillin-resistant Staphylococcus aureus isolates from the United States: establishing a national database. *J Clin Microbiol.* 2003;41:5113-20.

Miroux B, Walker JE. Over-production of proteins in *Escherichia coli*: mutant hosts that allow synthesis of some membrane proteins and globular proteins at high levels. *J Mol Biol*. 1996;260:289-98.

Monk IR, Shah IM, Xu M, Tan MW, Foster TJ. Transforming the untransformable: application of direct transformation to manipulate genetically *Staphylococcus aureus* and *Staphylococcus epidermidis*. mBio. 2012;3(2).

**Table S2** PCR primers

| **Primer** | **Function** | **5’ Primer Sequences** |
| --- | --- | --- |
| EJM 1 | Reverse primer for C terminal amplification of pSK-P2-*agrB* (primer pair EJM 1and 4) | TTTTAAGTCCTCCTTAATAAAGAAAATAG |
| EJM 2 | Forward primer for amplification of C terminal Large BiT (Primer pair EJM 1 and 2) | tctttattaaggaggacttaaaaGGCTCATCAGGTGGTGGC |
| EJM 3 | Reverse primer for amplification of C terminal Large BiT (Primer pair EJM 1 and 2) | ggaattcccggggatcctcaTCACGAGTTAATGGTCACACG |
| EJM 4 | Forward primer C terminal amplification of pSK-P2-*agrB* (primer pair EJM 1and 4) | TGAGGATCCCCGGGAATTC |
| EJM 5 | Forward primer for C terminal amplification of C terminal Small BiT (primer pair EJM 5 and 6) | tctttattaaggaggacttaaaaAGCGGGTCGTCTGGCGGT |
| EJM 6 | Reverse primer for C terminal amplification of C terminal Small BiT (primer pair EJM 5 and 6) | ggaattcccggggatcctcaTCAAAGAATTTCCTCAAACAGTCTATAG  CCTGTAACGC |
| EJM 7 | Reverse primer for N terminal amplification of pSK-P2-*agrB* (primer pair EJM 7 and 10) | TTTTACACCACTCTCCTC |
| EJM 8 | Forward primer for amplification of N terminal Large BiT (Primer pair EJM 8 and 9) | gtgaggagagtggtgtaaaaATGGTATTTACGCTTGAGGATTTT  GTCGGCG |
| EJM 9 | Reverse primer for amplification of N terminal Large BiT (Primer pair EJM 8 and 9) | attttattatcaaaataattcaaGCCGCTCGATCCGCCACC |
| EJM 10 | Forward primer N terminal amplification of pSK-P2-*agrB* (primer pair EJM 1and 4) | TTGAATTATTTTGATAATAAAATTGACC |
| EJM 11 | Forward primer for N terminal amplification of C terminal Small BiT (primer pair EJM 11 and 12) | gtgaggagagtggtgtaaaaATGGTTACAGGCTATAGACTGTTTGAA  GAGATTCTTGG |
| EJM 12 | Reverse primer for N terminal amplification of C terminal Small BiT (primer pair EJM 11 and 12) | attttattatcaaaataattcaaCCCGCCTGAGCTGCCTCC |
| EJM 55 | Forward primer confirming integration at *attB2* site | CATACTACATATCAACGAATCA |
| EJM 56 | Reverse primer confirming integration at *attB2* site | ATGGGTGGTAAAACACAAATTTC |
| IB1 | Forward primer amplifying AgrB1 | gccatcaccatcatcaccacTTGAATTATTTTGATAATAAAATTGACC |
| IB2 | Reverse primer amplifying AgrB1 | ctcgaattcggatcctggctTCATTTTAAGTCCTCCTTAATAAAG |
| IB3 | Forward primer amplifying pCDF duet-1 | AGCCAGGATCCGAATTCG |
| IB4 | Reverse primer amplifying pCDF duet-1 | GTGGTGATGATGGTGATG |
| EJM 307 | Mutational forward primer incorporating HiBiT at the N terminus of AgrB | GTTTAACTTTAATAAGGAGATATACCATGGTGAGCGGCTGGCGCCTGTT  CAAGAAGATTAGCGGCAGCAGCTTGAATTATTTTGATAATAAAATTGACC |
| EJM 308 | Mutational reverse primer incorporating HiBiT at the N terminus of AgrB | AAAATTATTTCTACAGGGGAATTGTTATC |
| EJM 309 | Mutational forward primer incorporating HiBiT at the C terminus of AgrB | CTATTTTCTTTATTAAGGAGGACTTAAAAAGCGGCAGCAGCGTGAGCGGC  TGGCGCCTGTTCAAGAAGATTAGCTGAATACATTATTTAACTTATTTTTTG |
| EJM 310 | Mutational reverse primer incorporating HiBiT at the C terminus of AgrB | GTAATAATGTAATAGCTTCTATTATGATGC |
| EJM 380 | Inverse amplification of pSK-P2-NL*agrB* (primer pair 380 and 387) | GCCGCTCGATCCGCCACCACC |
| EJM 381 | Amplification of *agrD* for N terminal Large BiT (primer pair EJM 381 and 382) | ggtggtggcggatcgagcggcATGAATACATTATTTAACTTA |
| EJM 382 | Amplification of *agrD* for N terminal Large BiT (primer pair EJM 381 and 382) | ggaattcccggggatcctcaTTATTCGTGTAATTGTGTT |
| EJM 383 | Inverse amplification of pSK-P2-NS*agrB* (primer pair 380 and 390) | CCCGCCTGAGCTGCCTCCTCC |
| EJM 384 | Amplification of *agrD* for N terminal Small BiT (primer pair EJM 384 and 382) | ggaggaggcagctcaggcgggATGAATACATTATTTAACTTA |
| EJM 385 | Amplification of *agrD* for C terminal Large BiT or Small BiT (primer pair EJM 385 and 386 or 389) | cagtgaggagagtggtgtaaaaATGAATACATTATTTAACTTATTT |
| EJM 386 | Amplification of *agrD* for C terminal Large BiT (primer pair EJM 385 and 386) | cgccaccacctgatgagccTTCGTGTAATTGTGTTAATTC |
| EJM 387 | Inverse PCR amplification of pSK-P2-*agrBCL* forward primer (primer pair EJM 387 and 388) | GGCTCATCAGGTGGTGGCG |
| EJM 388 | Inverse PCR amplification of pSK-P2-*agrBCL* reverse primer (primer pair EJM 387 and 388) | TTTTACACCACTCTCCTCACTG |
| EJM 389 | Amplification of *agrD* for C terminal Small BiT (primer pair EJM 389 and 385) | ctccaccgccagacgacccgcTTCGTGTAATTGTGTTAATTCTTTTG |
| EJM 390 | Inverse PCR amplification of pSK-P2-*agrBCS* reverse primer (primer pair EJM 390 and 388) | GCGGGTCGTCTGGCGGTGGAG |
| EJM 920 | Amplification of P2-AgrB (*Hind* III) forward | GTCAAA**AAGCTT**CTATTTTCCATCACATCTC |
| EJM 921 | Amplification of P2-AgrB (*Bam H*I) reverse | GTCAAA**GGATCC**TCATTTTAAGTCCTCCTTAATAAAG |

**Lower** **case** represents 5’ overhangs complementary to the vector sequence, **bold** indicates incorporated restriction sites and **underlined text** represents introduced HiBiT tag.

**Table S3**. *S. aureus* SH100 strains used for split luciferase assays to investigate AgrB-AgrB interactions. For these experiments one ectopic copy of *agrB* was integrated into the chromosomal *attB* site and the second copy was introduced on a plasmid. In each strain, the AgrBs were fused to either the luciferase LgBiT or the SmBiT sub-unit to generate AgrB protein fusions at either the N- or C-terminal or vice versa to ensure all possible combinations.

| ***S. aureus*** | **AgrB-AgrB Split Luciferase Combinations** |
| --- | --- |
| attB2:: SK-P2-*agrB***CL** p*agr*B**NL** | LgBiT fused to C-terminus of chromosomal AgrB; LgBit fused to N-terminus of plasmid AgrB |
| attB2:: SK-P2-*agrB***CL** p*agr*B**NS** | LgBiT fused to C-terminus of chromosomal AgrB; SmBiT fused to N-terminus of plasmid AgrB |
| attB2:: SK-P2-*agrB***CL** p*agr*B**CL** | LgBiT fused to C-terminus of chromosomal AgrB; LgBit fused to C-terminus of plasmid AgrB |
| attB2:: SK-P2-*agrB***CL** p*agr*B**CS** | LgBiT fused to C-terminus of chromosomal AgrB; SmBit fused to C-terminus of plasmid AgrB |
| attB2:: SK-P2-*agrB***NL** p*agr*B**NL** | LgBiT fused to N-terminus of chromosomal AgrB; LgBit fused to N-terminus of plasmid AgrB |
| attB2:: SK-P2-*agrB***NL** p*agr*B**CS** | LgBiT fused to N-terminus of chromosomal AgrB; SmBit fused to C-terminus of plasmid AgrB |
| attB2:: SK-P2-*agrB***NL** p*agr*B**CL** | LgBiT fused to N-terminus of chromosomal AgrB; LgBit fused to C-terminus of plasmid AgrB |
| attB2:: SK-P2-*agrB***NL** p*agr*B**NS** | LgBiT fused to N-terminus of chromosomal AgrB; SmBit fused to N-terminus of plasmid AgrB |
| attB2:: SK-P2-*agrB***NS** p*agr*B**NL** | SmBiT fused to N-terminus of chromosomal AgrB; LgBit fused to N-terminus of plasmid AgrB |
| attB2:: SK-P2-*agrB***NS** p*agr*B**NS** | SmBiT fused to N-terminus of chromosomal AgrB; SmBit fused to N-terminus of plasmid AgrB |
| attB2:: SK-P2-*agrB***NS** p*agr*B**CL** | SmBiT fused to N-terminus of chromosomal AgrB; LgBit fused to C-terminus of plasmid AgrB |
| attB2:: SK-P2-*agrB***NS** p*agr*B**CS** | SmBiT fused to N-terminus of chromosomal AgrB; SmBit fused to C-terminus of plasmid AgrB |
| attB2:: SK-P2-*agrB***CS** p*agr*B**NL** | SmBiT fused to C-terminus of chromosomal AgrB; LgBit fused to N-terminus of plasmid AgrB |
| attB2:: SK-P2-*agrB***CS** p*agr*B**CL** | SmBiT fused to C-terminus of chromosomal AgrB; LgBit fused to C-terminus of plasmid AgrB |
| attB2:: SK-P2-*agrB***CS** p*agr*B**NS** | SmBiT fused to C-terminus of chromosomal AgrB; SmBit fused to N-terminus of plasmid AgrB |
| attB2:: SK-P2-*agrB***CS** p*agr*B**CS** | SmBiT fused to C-terminus of chromosomal AgrB; SmBit fused to C-terminus of plasmid AgrB |

**Table S4***. S. aureus* SH100 strains used for split luciferase assays to investigate AgrB-AgrD interactions. For these experiments one ectopic copy of *agrB* was integrated into the chromosomal *attB* site and the *agrD* gene was introduced on a plasmid. In each strain, AgrB and AgrD were fused to either the luciferase LgBiT or the SmBiT sub-unit to generate AgrB and AgrD protein fusions at either the N- or C-terminal or vice versa to ensure all possible combinations.

| ***S. aureus*** | **AgrB-AgrB Split Luciferase Combinations** |
| --- | --- |
| SH1000 attB2:: SK-P2-*agrB***CL** p*agr*D**NS** | LgBiT fused to C-terminus of chromosomal AgrB; SmBiT fused to N-terminus of plasmid AgrD |
| SH1000 attB2:: SK-P2-*agrB***CL** p*agr*D**CL** | LgBiT fused to C-terminus of chromosomal AgrB; LgBiT fused to C-terminus of plasmid AgrD |
| SH1000 attB2:: SK-P2-*agrB***CL** p**NL** | LgBiT fused to C-terminus of chromosomal AgrB; LgBiT fused to N-terminus of plasmid AgrD |
| SH1000 attB2:: SK-P2-*agrB***CL** p*agr*D**NL** | LgBiT fused to C-terminus of chromosomal AgrB; LgBiT fused to N-terminus of plasmid AgrD |
| SH1000 attB2:: SK-P2-*agrB***CL** p**NS** | LgBiT fused to C-terminus of chromosomal AgrB; SmBiT only on plasmid |
| SH1000 attB2:: SK-P2-*agrB***CS** p*agr*D**NL** | SmBiT fused to C-terminus of chromosomal AgrB; LgBiTfused to N-terminus of plasmid AgrD |
| SH1000 attB2:: SK-P2-*agrB***CS** p*agr*D**NS** | SmBiT fused to C-terminus of chromosomal AgrB; SmBit fused to N-terminus of plasmid AgrD |
| SH1000 attB2:: SK-P2-*agrB***CS** p**NL** | SmBiT fused to C-terminus of chromosomal AgrB; LgBiT only on plasmid |
| SH1000 attB2:: SK-P2-*agrB***CS** p*agr*D**CL** | SmBiT fused to C-terminus of chromosomal AgrB; LgBit fused to C-terminus of plasmid AgrD |
| SH1000 attB2:: SK-P2-*agrB***NL** p**NL** | LgBiT fused to N-terminus of chromosomal AgrB; LgBit only on plasmid |
| SH1000 attB2:: SK-P2-*agrB***NL** p*agr*D**NS** | LgBiT fused to N-terminus of chromosomal AgrB; SmBit fused to N-terminus of plasmid AgrD |
| SH1000 attB2:: SK-P2-*agrB***NL** p*agr*D**NL** | LgBiT fused to N-terminus of chromosomal AgrB; LgBiT fused to N-terminus of plasmid AgrD |
| SH1000 attB2:: SK-P2-*agrB***NL** p**NS** | LgBiT fused to N-terminus of chromosomal AgrB; SmBiT only plasmid |
| SH1000 attB2:: SK-P2-*agrB***NS** p**NL** | SmBiT fused to N-terminus of chromosomal AgrB; LgBiT only on AgrD |
| SH1000 attB2:: SK-P2-*agrB***NS** p*agr*D**NS** | SmBiT fused to N-terminus of chromosomal AgrB; SmBiT fused to N-terminus of plasmid AgrD |
| SH1000 attB2:: SK-P2-*agrB***NS** p**NS** | SmBiT fused to N-terminus of chromosomal AgrB; SmBiT only on plasmid |
| SH1000 attB2:: SK-P2-*agrB***NS** p*agr*D**CL** | LgBiT fused to C-terminus of chromosomal AgrB; SmBiT fused to N-terminus of plasmid AgrD |
| SH1000 attB2:: SK-P2-*agrB***NS** p*agr*D**NL** | SmBiT fused to N-terminus of chromosomal AgrB; LgBit fused to N-terminus of plasmid AgrD |

N.B. we were unable to construct the C-terminal SmBiT fusion to AgrD.


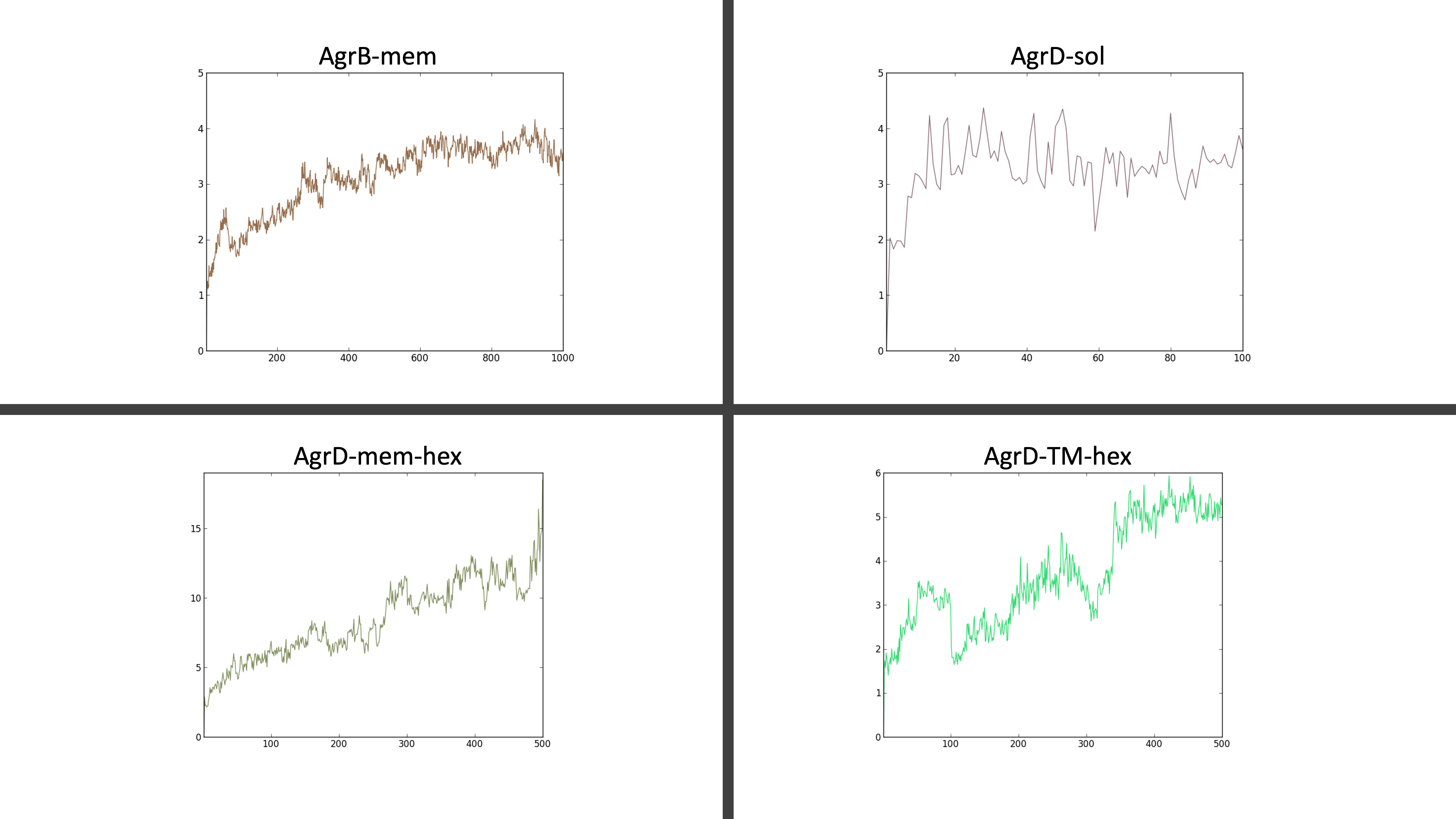


**Figure S1**: RMSD trajectory variation for AgrB in membrane and in complex with AgrD; AgrD in solution, associated with a membrane, integrated in a membrane, associated with an AgrB membrane and integrated in an AgrB membrane


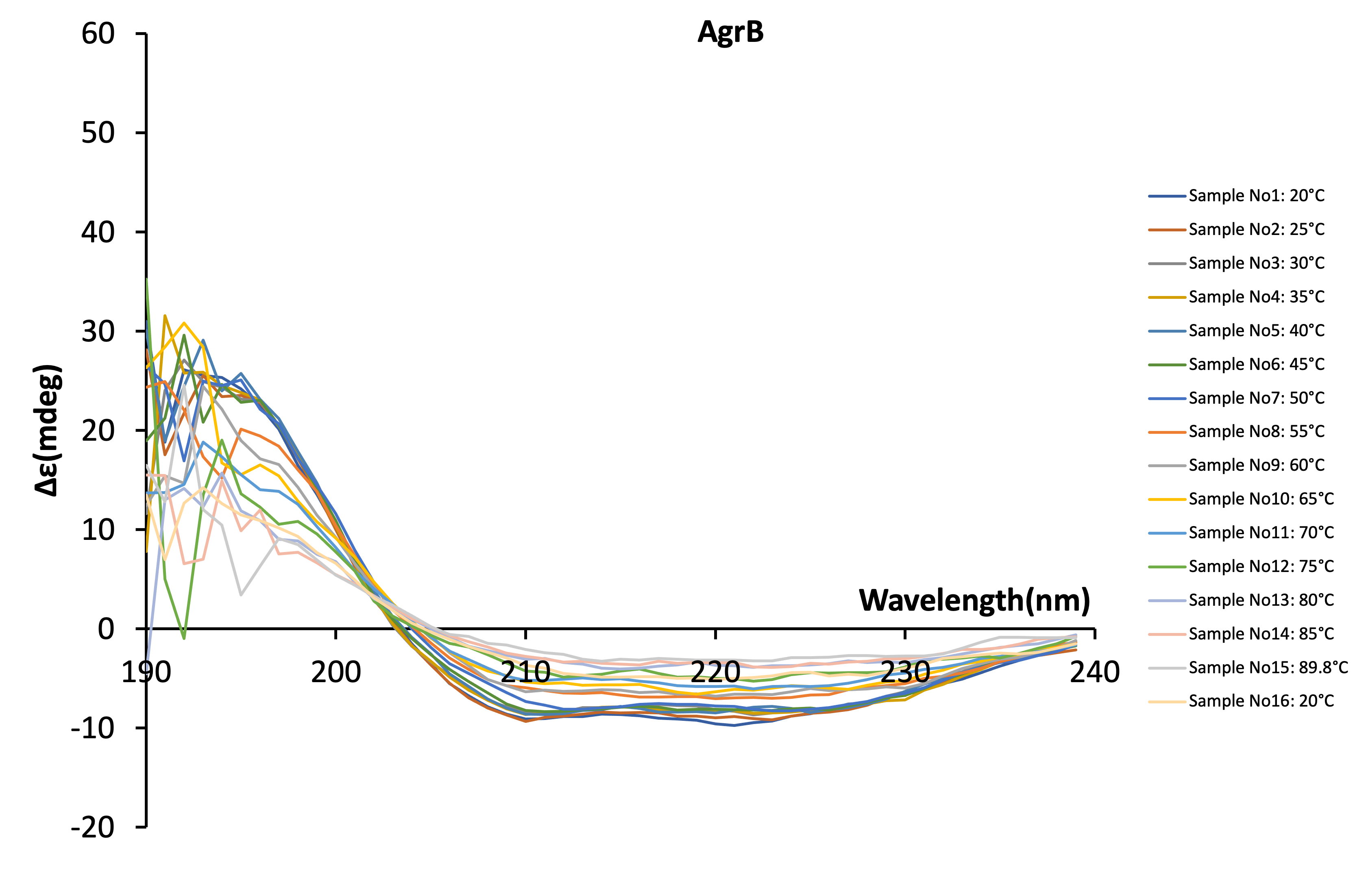


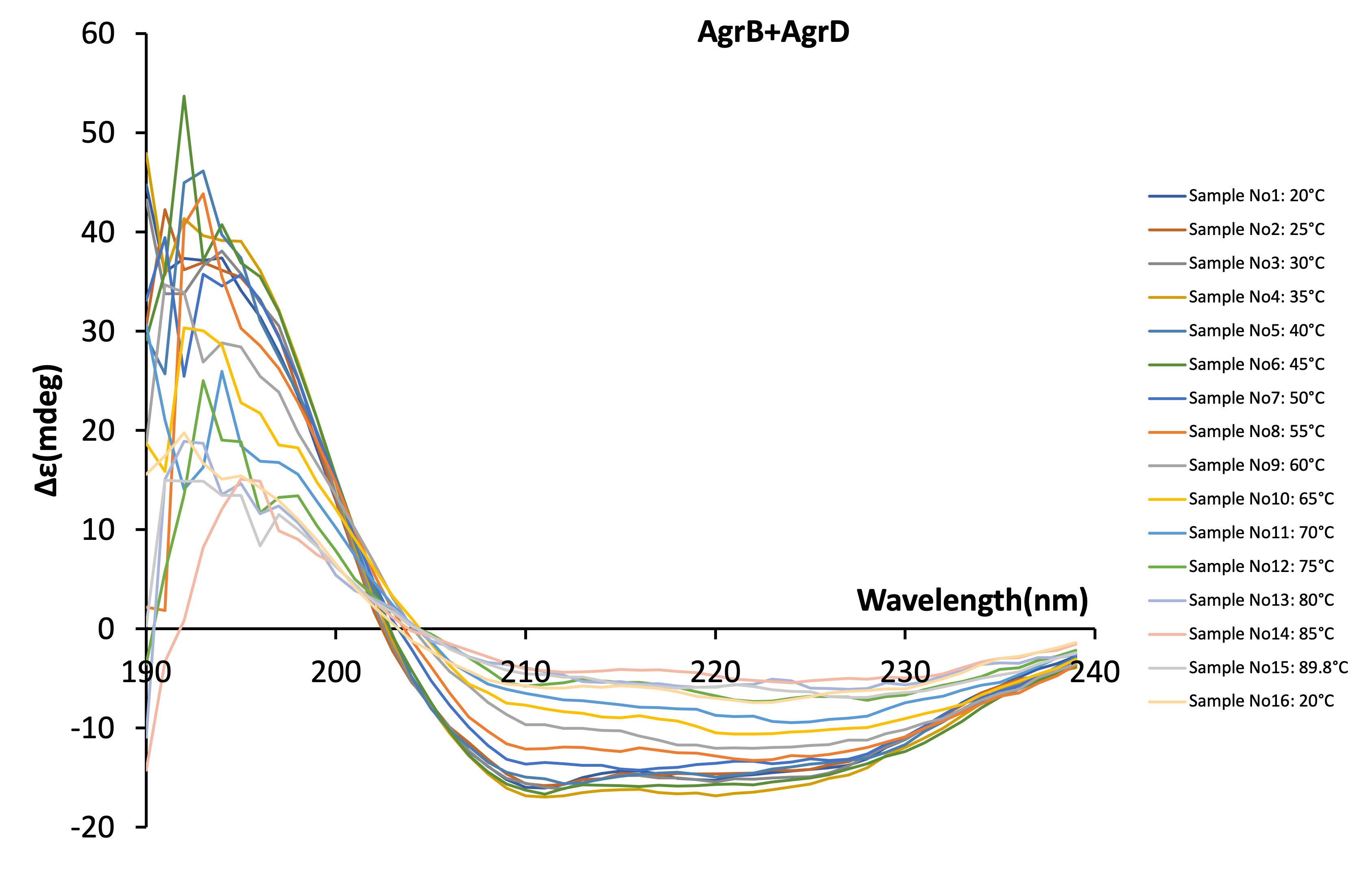


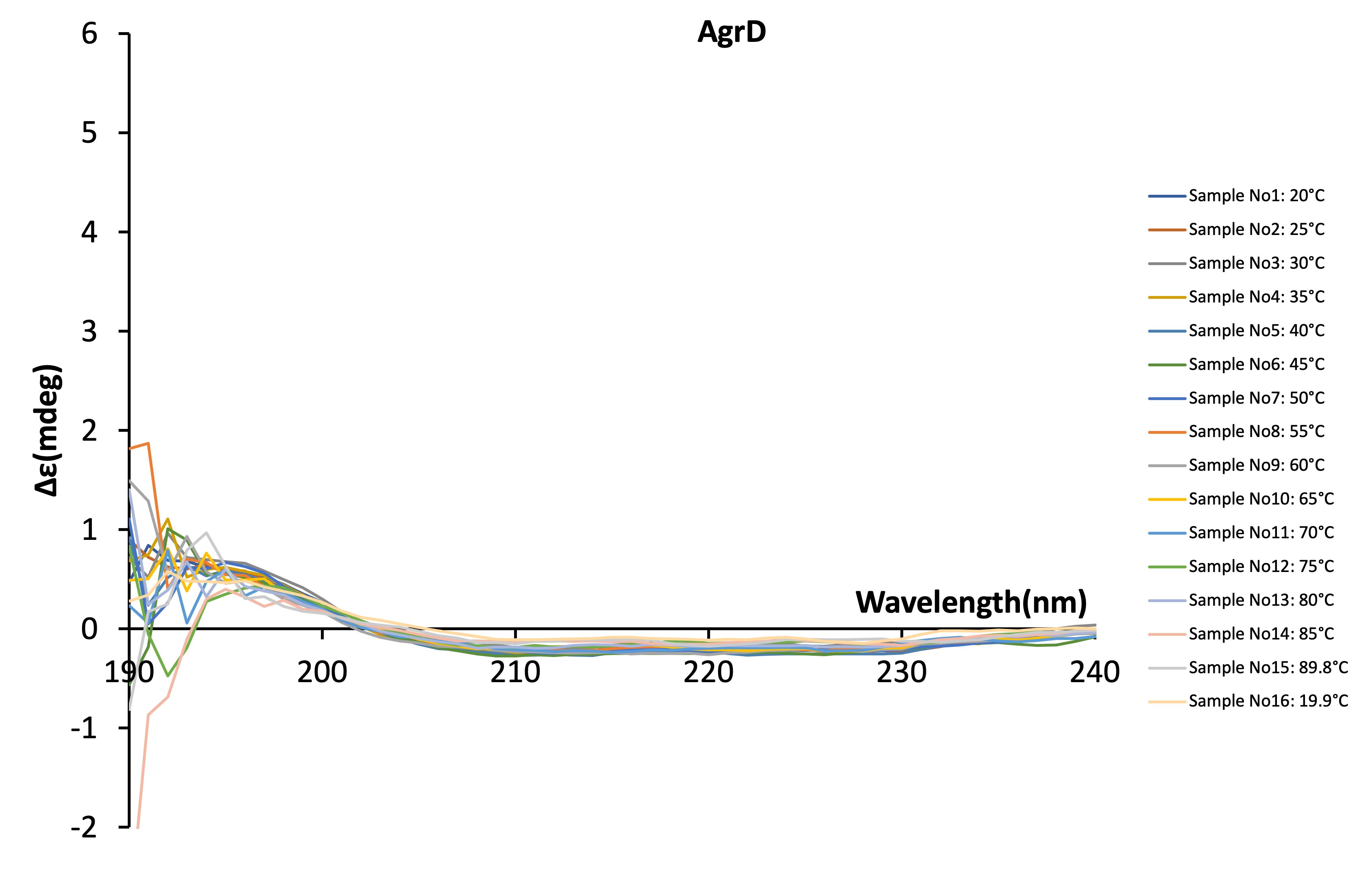


**Figure S2**: SRCD spectra collected during thermal denaturation of proteodetergent micelles containing AgrB (top); AgrB+AgrD (middle); and AgrD (bottom). The AgrD scale is zoomed ten-fold to show the weak peptide signal.


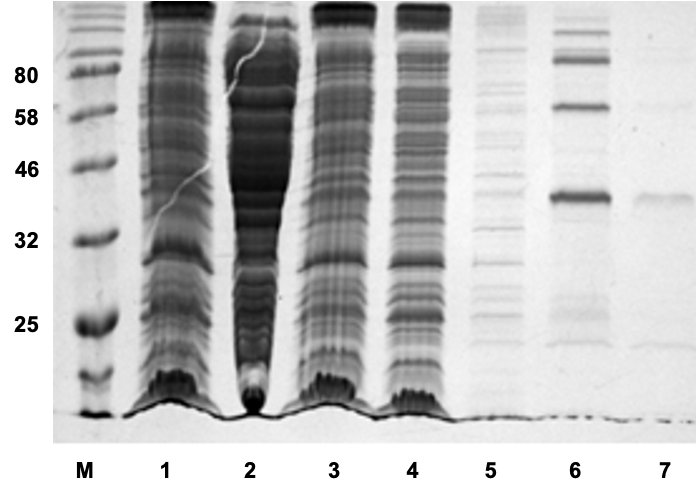


**Figure S3.** SDS-PAGE showing purification of recombinant AgrB from *E. coli* pCold-AgrB1 M, molecular mass markers (kDa), lane 1, soluble fraction, lane 2 cytoplasm, lane 3 DDM membrane extract, lane 4, column flow through, lane 5, column wash, lane 6, first imidazole elution; lane 7 second imidazole elution.
